# Temperature and Growth Selection Effects on Proliferation, Differentiation, and Adipogenic Potential of Turkey Myogenic Satellite Cells Through Frizzled-7-Mediated Wnt Planar Cell Polarity Pathway

**DOI:** 10.3389/fphys.2022.892887

**Published:** 2022-05-23

**Authors:** Jiahui Xu, Gale M. Strasburg, Kent M. Reed, Sandra G. Velleman

**Affiliations:** ^1^ Department of Animal Sciences, The Ohio State University, Wooster, OH, United States; ^2^ Department of Food Science and Human Nutrition, Michigan State University, East Lansing, MI, United States; ^3^ Department of Veterinary and Biomedical Sciences, University of Minnesota, St. Paul, MN, United States

**Keywords:** adipogenesis, growth selection, muscle, satellite cell, temperature, turkey

## Abstract

Satellite cells (SCs) are a heterogeneous population of multipotential stem cells. During the first week after hatch, satellite cell function and fate are sensitive to temperature. Wingless-type mouse mammary tumor virus integration site family/planar cell polarity (Wnt/PCP) signaling pathway is significantly affected by thermal stress in turkey pectoralis major (p. major) muscle SCs. This pathway regulates the activity of SCs through a frizzled-7 (Fzd7) cell surface receptor and two intracellular effectors, rho-associated protein kinase (ROCK) and c-Jun. The objective of the present study was to determine the effects of thermal stress, growth selection, and the Fzd7-mediated Wnt/PCP pathway on proliferation, myogenic differentiation, lipid accumulation, and expression of myogenic and adipogenic regulatory genes. These effects were evaluated in SCs isolated from the p. major muscle of 1-week faster-growing modern commercial (NC) line of turkeys as compared to SCs of a slower-growing historic Randombred Control Line 2 (RBC2) turkey line. Heat stress (43°C) increased phosphorylation of both ROCK and c-Jun with greater increases observed in the RBC2 line. Cold stress (33°C) had an inhibitory effect on both ROCK and c-Jun phosphorylation with the NC line showing greater reductions. Knockdown of the expression of *Fzd7* decreased proliferation, differentiation, and expression of myogenic regulatory genes: *myoblast determination factor-1* and *myogenin* in both lines. Both lipid accumulation and expression of adipogenic regulatory genes: *peroxisome proliferator-activated receptor-γ*, *CCAAT/enhancer-binding protein-β*, and *neuropeptide-Y* were suppressed with the *Fzd7* knockdown. The RBC2 line was more dependent on the Fzd7-mediated Wnt/PCP pathway for proliferation, differentiation, and lipid accumulation compared to the NC line. Thus, thermal stress may affect poultry breast muscle growth potential and protein to fat ratio by altering function and fate of SCs through the Fzd7-mediated Wnt/PCP pathway in a growth-dependent manner.

## 1 Introduction

Birds are homeotherms, and thus, maintain body temperature within a limited range (Yahav, 2015). Newly hatched poults have a poorly developed thermal regulatory system (Dunnington and Siegel, 1984; Modrey and Nichelmann, 1992), that limits their ability to cope with external thermal stress. For example, the body temperature of poults significantly changes with thermal stress (Shinder et al., 2007; Maman et al., 2019). Post-hatch thermal stress can affect muscle growth and structure by changing myofiber diameter (Piestun et al., 2017; Patael et al., 2019), capillary density (Joiner et al., 2014), and fat deposition (Zhang et al., 2012; Patael et al., 2019) particularly in the pectoralis major (p. major; breast) muscle. With intensive genetic selection for increased growth rate and breast muscle yield, modern faster-growing poultry lines produce more heat due to a greater metabolic rate (Konarzewski et al., 2000). In addition, faster-growing poultry also have a reduced ability to remove metabolic heat and byproducts like lactic acid due to decreased capillary supply in the p. major muscle (Hoving-Bolink et al., 2000; Joiner et al., 2014). Thus, faster-growing poultry lines have a lower tolerance to external heat stress (Berrong and Washburn, 1998; Chiang et al., 2008).

The number of myofibers is fixed by the time of hatch (Smith, 1963). Muscle growth after hatch occurs mainly through hypertrophy of existing myofibers mediated by the activity of adult myoblasts, satellite cells (SCs). Post-hatch myofiber hypertrophy proceeds through accretion of satellite cell (SC) nuclei to existing myofibers (Moss and Leblond, 1971; Cardasis and Cooper, 1975). During the period of SC peak mitotic activity, the first week after hatch in poultry (Mozdziak et al., 1994; Halevy et al., 2000), both proliferation and myogenic differentiation of SCs in the p. major muscle are highly responsive to environmental thermal stress (Halevy et al., 2001; Xu et al., 2021b). Thus, early-age hot and cold thermal stress has long-lasting effects on p. major muscle growth and structure (Piestun et al., 2017; Patael et al., 2019), in part, by altering the proliferation and myogenic activity of SCs.

As multipotential stem cells (Shefer et al., 2004), SCs also express adipogenic genes and produce lipid with appropriate extrinsic stimuli, including hot temperature (Harding et al., 2015; Patael et al., 2019; Xu et al., 2021a). Due to heterogeneity, SCs from the same myofiber type can contain different cell populations varying in proliferation and differentiation rate (McFarland et al., 1995), and adipogenic potential (Rossi et al., 2010). In *in vitro* studies, faster-proliferating SCs synthesize more lipid than slower-proliferating SCs (Rossi et al., 2010; Xu et al., 2021a). Selection for increased growth in turkeys has facilitated the conversion of the p. major muscle SCs to a faster-proliferating population (Velleman et al., 2000; Clark et al., 2016; Xu et al., 2021b) with increased lipid production (Velleman, 2014; Clark et al., 2017; Xu et al., 2021a). Heat stress further increases the lipid content in SCs, while it is suppressed under cold stress (Clark et al., 2017; Xu et al., 2021a). Increased lipid synthesis by SCs *in vivo* will result in increased intramuscular fat deposition (Velleman et al., 2014; Piestun et al., 2017; Patael et al., 2019), and alter protein to fat ratio in the poultry p. major muscle.

Changes in cellular function with response to thermal stress are mediated through signal transduction pathways. Results from transcriptome analysis showed that the expression of genes associated with wingless-type mouse mammary tumor virus integration site family (Wnt) signaling pathway was greatly altered by both hot and cold thermal stress in turkey p. major muscle SCs (Reed et al., 2017a). Among the most affected *Wnt* genes, *Wnt7a* expression was upregulated with heat stress, particularly in a growth-selected turkey line (Reed et al., 2017a). In mice, protein-ligand Wnt7a activates a non-canonical planar cell polarity (PCP) pathway in SCs through a cell surface receptor, frizzled-7 (Fzd7) (Le Grand et al., 2009; Von Maltzahn et al., 2012). A recent *in vitro* study by Risha et al. (2021) showed that both hot and cold thermal stress significantly altered the expression of *Wnt7a* and *Fzd7* in mouse myoblasts. Thus, in the turkey p. major muscle SCs, thermal stress may similarly function through the Wnt/PCP pathway and Fzd7 receptor.

Downstream branches of the Wnt/PCP pathway include but are not limited to: 1) ras homolog gene family member A (RhoA) (Strutt et al., 1997; Fanto et al., 2000) through its effector rho-associated protein kinase (ROCK) (Laumanns et al., 2009); and 2) ras-related C3 botulinum toxin substrate 1 (Rac1) (Eaton et al., 1996; Fanto et al., 2000) *via* its effector c-Jun (Weber et al., 2000). In mammalian skeletal muscle, the Wnt/PCP pathway promotes proliferation of SCs through the Fzd7 receptor and increases the SC pool stimulating myofiber hypertrophy resulting in increased skeletal muscle mass (Le Grand et al., 2009). The Fzd7-mediated Wnt-PCP pathway is also involved in the rearrangement of cytoskeleton protein, actin, during cell migration *via* the RhoA/ROCK signaling (Fortier et al., 2008; Kaucká et al., 2013; Asad et al., 2014; Wang et al., 2018). Migration is required for SC alignment before fusion to form multinucleated myotubes (Chazaud et al., 1998). In addition to migration, RhoA/ROCK signaling is also involved in regulating myogenic differentiation (Iwasaki et al., 2008) and adipogenic potential (Sordella et al., 2003; Bryan et al., 2005; Hosoyama et al., 2009) in mouse myoblasts. Another Wnt/PCP effector, c-Jun, is a cell proliferation activator that promotes cell cycle progression through G1 phase (Schreiber et al., 1999; Wisdom et al., 1999). In myogenic myoblasts (Daury et al., 2001) and SCs (Umansky et al., 2015; Hindi and Kumar, 2016), the primary function of c-Jun is to stimulate proliferation and inhibit myogenic differentiation. Thus, one of the objectives in the present study was to analyze the role of the Fzd7-mediated Wnt/PCP pathway in thermal stress-induced changes in the function and fate of SCs.

Although previous evidence showed the Wnt/PCP pathway is involved in proliferation (Le Grand et al., 2009), migration (Wang et al., 2018), and adipogenesis (Bryan et al., 2005) through its downstream effectors in mammalian SCs, the role of the Wnt/PCP pathway in regulating the function and fate of SCs in poultry during thermal stress has not been examined. Growth-selected turkeys display differential expression of *Wnt* genes in SCs compared to non-growth-selected turkeys (Reed et al., 2017a). With peak mitotic activity during the first week after hatch (Mozdziak et al., 1994; Halevy et al., 2000), the function and fate of turkey SCs are highly responsive to thermal stress (Xu et al., 2021a; Xu et al., 2021b) likely through the Wnt/PCP pathway. Thus, the objective of the present study was to determine the effects of thermal stress, growth selection, and the Fzd7-mediated Wnt/PCP pathway on proliferation, myogenic differentiation, lipid accumulation, and expression of myogenic and adipogenic regulatory genes in the p. major muscle SCs isolated from 1-week turkeys. To determine how the Fzd7-mediated Wnt/PCP pathway participates in regulating SC activity, *Fzd7* expression was knocked down with small interfering RNA (siRNA) in SCs isolated from modern commercial (NC) line turkeys and Randombred Control Line 2 (RBC2) turkeys representing slower-growing turkeys of the 1960s (Nestor et al., 1969). Alterations in SC function and fate will have long-term effects on the growth, structure, and protein to fat ratio of the poultry p. major muscle.

## 2 Materials and Methods

### 2.1 Satellite Cells

Satellite cells were previously isolated from the p. major muscle of 1-week-old RBC2 and NC turkeys following the method of Velleman et al. (2000). Only male turkeys were selected for cell isolation to avoid the confounding effect of sex (Velleman et al., 2000). All the SCs were passaged to a fourth pass and stored in liquid nitrogen until use.

### 2.2 Small Interfering RNA

Small interfering RNA targeting *Fzd7* (Gene bank ID: XM_010713460.1) was synthesized as a stealth siRNA duplex (Thermo Fisher Scientific, Waltham, MA, United States), and was designed using Invitrogen Block-iT siRNA designer (https://rnaidesigner.thermofisher.com/rnaiexpress/setOption.do?designOption=stealth&pid). The *Fzd7* siRNA targets the *Fzd7* open reading frame from 1522 to 1546 with the following sequence: sense strand: 5′-CCG GAC UUC ACA GUC UUC AUG AUC A-3′; anti-sense strand: 5′-UGA UCA UGA AGA CUG UGA AGU CCG G-3′. A stealth siRNA with 48% guanine and cytosine content was used as the negative control siRNA (Thermo Fisher Scientific).

To determine the knockdown efficiency of the *Fzd7* siRNA, p. major muscle SCs from each line were plated (18,000 cells per well) in 24-well gelatin-coated plates (Greiner Bio-One, Monroe, NC, United States) in 500 μl of Dulbecco’s Modified Eagle’s Medium (DMEM, Sigma-Aldrich, St. Louis, MO, United States) transfection medium supplemented with 10% chicken serum (Gemini Bio-Products, West Sacramento, CA, United States) and 5% horse serum (Gemini Bio-Products), and incubated at 38°C in a 95% air/5% CO_2_ incubator (Thermo Fisher Scientific) for 24 h allowing for cell attachment. After 24 h, cells in each well were transfected with 20 pmol/μl of either the *Fzd7* siRNA or the negative control siRNA with 1 μl of lipofectamine 2000 (Thermo Fisher Scientific) according to the manufacturer’s protocol. After 12 h of transfection, the medium was replaced with McCoy’s 5A growth medium (Sigma Aldrich) containing 10% chicken serum (Gemini Bio-Products), 5% horse serum (Gemini Bio-Products), 1% antibiotics-antimycotics (Corning, Corning, NY, United States), and 0.1% gentamicin (Omega Scientific, Tarzana, CA, United States) for 72 h of proliferation with the medium changed every 24 h. At 72 h post transfection, cells were removed from the incubator and total RNA was extracted for gene expression analysis by Real-Time Quantitative PCR (RT-qPCR) as described in Section 2.8. The transfection experiment and RT-qPCR was independently repeated twice to confirm knockdown efficiency of the *Fzd7* siRNA.

### 2.3 Western Blot Analysis

The RBC2 and NC line SCs were plated in 24-well plates (15,000 cells per well) in a DMEM plating medium containing 10% chicken serum, 5% horse serum, 1% antibiotics-antimycotics, and 0.1% gentamicin. The plated cells were allowed to attach for 24 h while incubated at 38°C in a 95% air/5% CO_2_ incubator. After 24 h, the plating medium was replaced with the growth medium. Satellite cells from both lines were then randomly assigned to proliferate at 33°C, 38°C, or 43°C for 72 h, with the growth medium changed every 24 h. After 72 h of proliferation, the growth medium was replaced with a DMEM differentiation medium containing 3% horse serum, 1% antibiotics-antimycotics, 0.1% gentamicin, 0.1% gelatin, and 1 mg/ml bovine serum albumin (BSA, Sigma Aldrich). The differentiation medium was changed every 24 h. At 24-h intervals during differentiation, one plate in each group was removed from incubation and rinsed with phosphate buffered saline (PBS, 171 mM NaCl, 3.35 mM KCl, 1.84 mM KH_2_PO_4_, and 10 mM Na_2_HPO_4_, pH7.08) for protein extraction.

For *Fzd7* siRNA transfection, cell culture and transfection procedures were the same as described in Section 2.3 until 72 h of proliferation. At 72 h of proliferation, the growth medium was replaced with differentiation medium, and incubation continued with the differentiation medium changed every 24 h. At 48 h of differentiation, all plates were removed, rinsed with PBS and total protein immediately extracted.

For protein extraction, 100 μl per well of ice-cold protein extraction buffer [50 mM Tris–HCl, 1% Nonidet P-40, 0.5% sodium deoxycholate, 0.1% sodium dodecyl sulphate (SDS), 150 mM NaCl, 1 mM EDTA, 1 mM Na_3_VO_4_, and protease and phosphatase inhibitors (Thermo Fisher Scientific)] was added to the 24-well plates and incubated for 15 min on ice. Cells were scraped from each well, and the lysate was incubated on ice for another 15 min. A syringe with a 26 G needle was used to aspirate the cell lysate. The cell lysate was centrifuged at 10,000 rpm for 15 min at 4°C, and supernatant was collected. Protein concentration was measured using the Bradford method (Bradford, 1976) and samples were adjusted to equal concentration. A denaturation buffer containing 0.1 M tris-HCl, 10% glycerol, 1% β-mercaptoethanol, 0.1% bromophenol blue, and 1% sodium dodecyl sulfate (SDS) was added to each protein sample before boiling for 10 min. The denatured samples (30 μg/well) and a pre-stained protein molecular weight standard (Thermo Fisher Scientific) were separated using 8% sodium dodecyl sulfate-polyacrylamide gel electrophoresis (SDS-PAGE) with 40 mA of constant current in a running buffer (25 mM Tris, 200 mM glycine, and 10% SDS, pH 8.3) according to the method of Laemmli (1970). Separated proteins were transferred from the SDS-PAGE to a polyvinylidene difluoride (PVDF) membrane at 200 mA in a transfer buffer containing 25 mM tris, 192 mM glycine, and 20% methanol for 2.5 h on ice. The PVDF membrane was blocked with a blocking buffer (5% non-fat milk, 20% Tween-20, 20 mM Tris-HCl, 500 mM NaCl, pH 7.5) for 60 min at room temperature, then, incubated overnight with a primary antibody diluted in blocking buffer at 4°C. Primary antibodies included: rabbit anti-ROCK1 (1:1000 dilution, Abcam, Waltham, MA, United States), rabbit anti-phospho-ROCK1 at Thr455/Ser456 (1:700 dilution, Bioss Antibodies, Woburn, MA, United States), rabbit anti-c-Jun (1:700 dilution, MyBioSource, San Diego, CA, United States), rabbit anti-phospho-c-Jun at Ser73 (1:700 dilution, Cell Signaling Technology, MA, United States), and rabbit anti-β-actin (1:1,000 dilution, Cell Signaling Technology). After incubation with primary antibody, the PVDF membrane was gently agitated for 10 min in a washing buffer (20% Tween-20, 20 mM Tris-HCl, 500 mM NaCl, pH 7.5) for three times. The PVDF membrane was then incubated for 2 h at room temperature in a horseradish peroxidase-conjugated goat anti-rabbit secondary antibody (1:1000 dilution, Cell Signaling Technology) in the blocking buffer. After gentle agitation the PVDF membrane in washing buffer three times, a chemiluminescent substrate (Thermo Fishier Scientific) was used to visualize target protein bands on a Bio-Rad ChemiDoc XRS imaging system (Bio-Rad, Hercules, CA, United States). The membrane was re-stripped using a western blot stripping buffer (Thermal Fishier Scientific) before incubating with other primary antibodies as described above. Density of the band β-actin was used to normalize each target protein. The ratio of phosphorylated protein to total protein was calculated from normalized band densities (Zhang et al., 2008). Fold change was calculated as the ratio of each treatment group divided by the calculated ratio of the control group. The western blot analysis was independently repeated in twice per treatment. Due to reduced cellular growth at 33°C and being able to isolate sufficient protein, the following number of replicate wells were used: 12 wells per cell line at 38°C and 43°C, 24 wells per cell line at 33°C.

### 2.4 Proliferation Assay

Cell culture and transfection procedures were the same as described in Section 2.2. During the 72 h of proliferation, one plate from each treatment group was removed from incubator every 24 h and stored at −70°C until assayed.

Proliferation assay was conducted according to the method of McFarland et al. (1995). All plates were removed from −70°C and thawed at room temperature for 15 min, 200 μl of 0.05% trypsin-EDTA (Thermo Fisher Scientific) in 10 mM Tris, 2 M NaCl, and 1 mM EDTA (TNE) was added to each culture well and incubated at room temperature for 7 min. The plates were then returned to −70°C overnight. After thawing the plates for 15 min at room temperature, 1.8 ml of TNE buffer containing 0.2% (1 mg/ml) Hoechst dye (Sigma-Aldrich) was added to each well, and the plates were gently agitated for 2 h at room temperature. DNA-incorporated Hoechst dye was measured using a Fluoroskan Ascent FL plate reader (Thermo Fisher Scientific). A standard curve constructed using double-stranded calf thymus DNA (Sigma-Aldrich) to determine sample DNA concentration. The proliferation assay was repeated in two independent cultures with four replicate wells per treatment per culture.

### 2.5 Differentiation Assay

For transfection at the beginning of proliferation, 11, 000 cells were plated in each well of 48-well gelatin-coated plates in 500 μl of transfection medium and incubated at 38°C in a 95% air/5% CO_2_ incubator. After 24 h of attachment, cells in each well were transfected with 20 pmol/μl of either *Fzd7* siRNA or negative control siRNA with 0.5 μl of Lipofectamine 2000. After 12 h of transfection at 38°C, the transfection medium was replaced with growth medium, and cells were randomly assigned to a 33°C, 38°C, or 43°C incubator for 72 h, with the growth medium changed daily. After 72 h of proliferation, the growth medium was replaced with differentiation medium for 72 h of differentiation with the media changed every day. One plate from each treatment group was removed at 24 h of intervals, rinsed with PBS, and stored at −70°C for the differentiation assay.

For transfection at the beginning of differentiation (at 0 h of differentiation), 9,000 cells were plated in each well of 48-well gelatin-coated plates in plating medium at 38°C. After 24 h of attachment, plating medium was replaced with growth medium, and cells from both lines were randomly assigned to a 33°C, 38°C, or 43°C incubator for 72 h of proliferation. The growth medium was changed every 24 h. At 72 h of proliferation, the cells were transfected with 20 pmol/μl of either the *Fzd7* siRNA or the negative control siRNA with 0.5 μl Lipofectamine 2000 per well. After 12 h of transfection, the growth medium was replaced with differentiation medium for 72 h of differentiation. Differentiation medium was changed every 24 h. One plate from each treatment group was removed every 24 h, rinsed with PBS and stored at −70°C for the differentiation assay.

Satellite cell differentiation was determined by measuring creatine kinase activity using a modified method of Yun et al. (1997). All plates were removed from −70°C and thawed at room temperature for 15 min, and 500 μl of creatine kinase buffer [20 mM glucose (Thermo Fisher Scientific), 20 mM phosphocreatine (Calbiochem, San Diego, CA, United States), 10 mM mg acetate (Thermo Fisher Scientific), 10 mM adenosine monophosphate (Sigma Aldrich), 1 mM adenosine diphosphate (Sigma Aldrich), 1 Unit (U)/ml glucose-6-phosphate dehydrogenase (Worthington Biochemical), 0.5 U/ml hexokinase (Worthington Biochemical, Lakewood, NJ, United States), 0.4 mM thio-nicotinamide adenine dinucleotide (Oriental Yeast Co., Tokyo, Japan), 1 mg/ml BSA, to 0.1 M glycylglycine (PH = 7.5)] was added to each well including the standard curve wells containing creatine phosphokinase with concentrations from 0 to 140 milliunits/well (mU/well, Sigma-Aldrich). The optical density of each well was measured at a wavelength of 405 nm using a BioTek ELx800 (BioTek, Winooski, VT, United States) plate reader. The differentiation assay was repeated in two independent cultures with five wells per treatment per culture.

### 2.6 Myotube Measurement

Cell culture and transfection procedures were as described in Section 2.5. At 48 h of differentiation, photomicrographs of SCs and myotubes in each treatment group were taken with an Olympus IX70 fluorescence microscope equipped with a QImaging Retiga Exi Fast digital camera (Qimaging, Surrey, BC, Canada) and CellSens software (Olympus America, Center Valley, PA, United States). The diameter of the myotubes was measured using Image Pro Software (Media Cybernectics, Silver Spring, MD, United States). A total of 100 measurements were taken per treatment with measurements independently repeated in two cultures with five replicate wells per treatment per culture.

### 2.7 Lipid Content Measurement

Cell culture and transfection procedures were the same as described in Section 2.2 during proliferation. At 72 h of proliferation, growth medium was replaced with differentiation medium. Differentiation medium was changed every 24 h for the 72 h of differentiation. One plate from each treatment group was collected at 24 h intervals during the 72 h of proliferation and 72 h of differentiation for AdipoRed assay.

Lipids were stained with AdipoRed lipid fluorochrome (Lonza, Walkersville, MD, United States). In brief, after removing the medium, each well was rinsed twice with PBS. Another 1 ml of PBS containing 30 μl of AdipoRed was added to each well with the cultured cells and one blank well without any cells in. After incubating for 15 min at room temperature, the AdipoRed optical density (OD) of each well was measured in the Fluoroskan Ascent FL plate reader at a wavelength of 485 nm. The final OD of each well was the OD of each well minus the measured OD of the blank well. The AdipoRed assay was independently repeated in two cultures with four replicate wells per treatment per culture.

### 2.8 Gene Expression Analysis

Myogenic regulatory genes measured in this study include myoblast determination factor 1 (*MyoD*) and myogenin (*MyoG*) with the former a proliferation marker (Yablonka-Reuveni and Rivera, 1994) and the latter a myogenic differentiation marker (Brunetti and Goldfine, 1990; Hasty et al., 1993). Adipogenic regulatory genes examined included peroxisome proliferator activated receptor-gamma (*PPARγ*) (Rosen et al., 1999), neuropeptide-Y (*NPY*) (Baker et al., 2009), and CCAAT/enhancer-binding protein-beta (*C/EBPβ*) (Clarke et al., 1997).

Total RNA from each sample was extracted using RNAzol (Molecular Research Center, OH, United States) and the concentration of each sample was quantified by a spectrophotometer (NanoDrop™ ND-1000, Thermo Fisher Scientific). Reverse transcription was performed with Moloney Murine Leukemia Virus Reverse Transcriptase (M-MLV; Promega, WI, United States) to produce cDNA from total RNA. Real-Time Quantitative PCR (RT-qPCR) was conducted with DyNAmo Hot Start SYBR Green qPCR kit (Thermo FisherScientific). Information of all the primers is listed in Table 1. Primers for *C/EBPβ*, *MyoD, MyoG, NPY*, *PPARγ*, and an internal control gene glyceraldehyde-3-phosphate dehydrogenase (*GAPDH*) were previously designed, and the specificity was confirmed in this lab as reported by Clark et al. (2017), Clark et al. (2018). Primers for *Fzd7* were designed using primer-BLAST tool (https://www.ncbi.nlm.nih.gov/tools/primer-blast/), and the specificity of the *Fzd7* primers was confirmed by DNA sequencing the PCR product (Molecular and Cellular Imaging Center, The Ohio State University, Wooster, OH). The RT-qPCR reaction was performed in a DNA Engine Opticon 2 real-time machine (Bio-Rad) and included: 1) denaturation at 94°C for 15 min; 2) amplification for 35 cycles with each cycle including denaturation for 30 s at 94°C, annealing for 30 s at 55°C (*Fzd7*, *GAPDH*, *NPY*, and *PPARγ*), at 58°C (*MyoD* and *MyoG*), or at 60°C (*C/EBPβ*), and elongation for 30 s at 72°C; and 3) final elongation at 72°C for 5 min. A standard curve of each gene was generated using serial dilutions of purified PCR products (Liu et al., 2006). Arbitrary concentrations from 1 to 100,000 were assigned to each serial dilution. An arbitrary molar concentration of each amplified sample was calculated according to the threshold cycle and was normalized by *GAPDH*. The RT-qPCR for each gene was independently repeated in two cultures with twelve wells per treatment per culture.

**TABLE 1:** Primer sequences for real-time quantitative polymerase chain reaction.

Primer	Sequence	Product size	GenBank accession number
*MyoD* ^a^	5′-GAC GGC ATG ATG GAG TAC AG-3′ (forward)	201 bp^h^	AY641567.1
	5′-AGC TTC AGC TGG AGG CAG TA-3′ (reverse)		
*MyoG* ^b^	5′-CCT TTC CCA CTC CTC TCC AAA-3′ (forward)	175 bp	AY560111.3
	5′-GAC CTT GGT CGA AGA GCA ACT-3′ (reverse)		
*Fzd7* ^c^	5′-CAC CGG CTT CTC CTT TTC TTG-3′ (forward)	207 bp	XM_010713460.1
	5′-ACA GTA AAG AGG GTG GAC GC-3′ (reverse)		
*PPARγ* ^d^	5′-CCA CTG CAG GAA CAG AAC AA-3′ (forward)	249 bp	XM_010718432.1
	5′-CTC CCG TGT CAT GAA TCC TT-3′ (reverse)		
*C/EBPβ* ^e^	5′-GCA CAG CGA CGA GTA CAA G-3′ (forward)	82 bp	XM_003212165.2
	5′-GTT GCG CAT TTT GGC TTT GTC-3′ (reverse)		
*NPY* ^f^	5′-CCC AGA GAC ACT GAT CTC AGA C-3′ (forward)	76 bp	XM_010712774.3
	5′-AGG GTC TTC AAA CCG GGA TCT-3′ (reverse)		
*GAPDH* ^g^	5′-GAG GGT AGT GAA GGC TGC TG-3′ (forward)	200 bp	U94327.1
	5′-CCA CAA CAC GGT TGC TGT AT-3′ (reverse)		

a *MyoD*, myogenic determination factor-1.

b *MyoG*, myogenin.

c *Fzd7*, frizzled-7.

d *PPARγ*, peroxisome proliferator-activated receptor gamma.

e *C/EBPβ*, CCAAT/enhancer-binding protein beta.

f *NPY*, neuropeptide Y.

g *GAPDH*, glyceraldehyde-3-phosphate dehydrogenase.

h bp, number of base pairs.

### 2.9 Statistical Analysis

Data from proliferation, differentiation, and AdipoRed assays, myotube measurement, and gene expression analysis were analyzed as a mixed model at each sampling time in SAS (SAS 9.4, SAS Institute INC., NC, United States). Two fixed effects of temperature and cell line, an interaction effect between temperature and cell line, and a random effect of repeat experiment were included in the model. The statement of lsmean in the MIXED procedure was used to determine each mean value and the standard error of the mean (SEM). Differences between each mean were separated with the Pdiff option. Line effect within each treatment group and temperature effect within each cell line was determined with the SLICE option at each sampling time. For AdipoRed assay, the REG procedure was used to evaluate the linear relationship between sampling times and the OD of AdipoRed for each cell line in each treatment group. Difference in the linear response was determined with the contrast statement. *p* ≤ 0.05 was considered as statistically significant.

## 3 Results

### 3.1 Effects of Thermal Stress and Growth Selection on Rho-Associated Protein Kinase and c-Jun Phosphorylation

**FIGURE 1:**
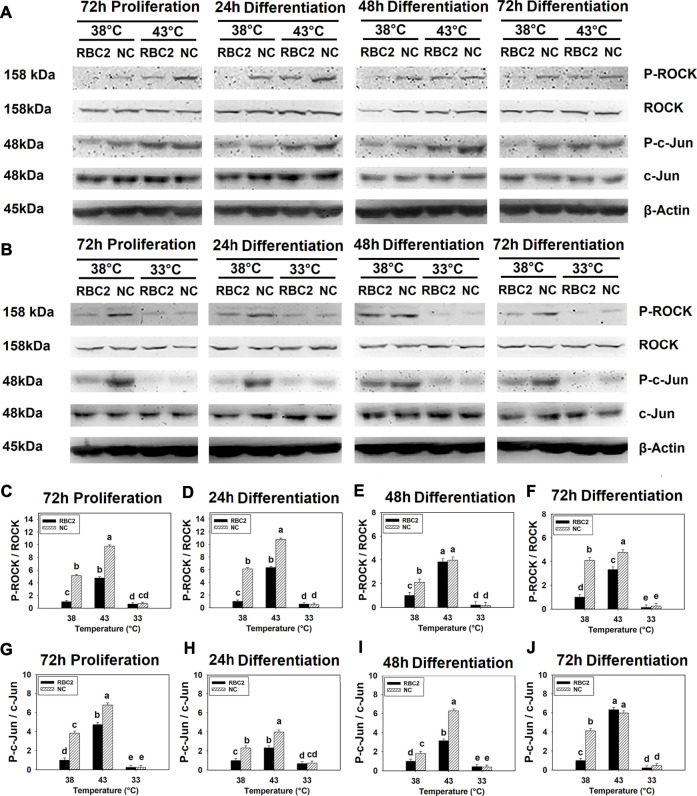
Effect of hot and cold thermal stress on the phosphorylation of rho-associated protein kinase (ROCK) and c-Jun in satellite cells (SCs). **(A)** Western blots of unphosphorylated and phosphorylated ROCK and c-Jun, and an internal control β-actin in the Randombred Control Line 2 (RBC2) and modern commercial (NC) line SCs cultured at 38°C or 43°C and determined at 72 h of proliferation (also represents 0 h of differentiation) and 24, 48, and 72 h of differentiation. **(B)** Western blots of unphosphorylated and phosphorylated ROCK and c-Jun, and an internal control β-actin in the RBC2 and NC line SCs cultured at 38°C or 33°C and determined at 72hP, 24hD, 48hD, and 72hD. Molecular weight is on the left and protein name is on the right side of each figure, respectively. The ratio of phosphorylated to unphosphorylated ROCK band density was calculated at 72 h of proliferation **(C)**, 24 h **(D)**, 48 h **(E)**, and 72 h **(F)** of differentiation. The ratio of phosphorylated to unphosphorylated c-Jun band density was calculated at 72 h of proliferation **(G)**, 24 h **(H)**, 48 h **(I)**, and 72 h **(J)** of differentiation. Each graph bar represents a mean ratio, and each error bar represents a standard error of the mean. Mean values with different letters are significantly different (*p* ≤ 0.05).

Phosphorylation of ROCK showed a significant interaction effect between temperature and cell line (*p* < 0.001) at all sampling times (Figures 1A–F). In the RBC2 and NC line SCs, heat stress (43°C) increased ROCK phosphorylation, while cold stress (33°C) showed a significant inhibitory effect (*p* < 0.001). At both 38°C and 43°C, ROCK phosphorylation was greater in the NC line than RBC2 line at 72 h of proliferation, and 24 and 72 h of differentiation (*p* < 0.001). During the cold stress of 33°C, line effects were not significant at any sampling times.

Similarly, a significant interaction effect (*p* < 0.001) between temperature and cell line was observed for c-Jun phosphorylation at all sampling times (Figures 1A,B,G–J). Phosphorylation in both lines increased at 43°C but decreased at 33°C compared to the control temperature of 38°C (*p* < 0.001). The NC line showed greater c-Jun phosphorylation at both 38°C and 43°C from 72 h of proliferation to 48 h of differentiation (*p* < 0.001). A significant line effect was not observed at 33°C at any sampling time.

### 3.2 Effect of Frizzled-7 Knockdown, Thermal Stress, and Growth Selection on Rho-Associated Protein Kinase and c-Jun Phosphorylation

Phosphorylation profiles of both ROCK and c-Jun during 72 h of differentiation in the absence of *Fzd7* knock down are shown in Supplementary Figure S1 *.* Phosphorylation of both ROCK and c-Jun peaked at 24 h of differentiation in both lines at 38°C and 43°C (Supplementary Figures S1A–F). At 33°C, the NC line showed maximal phosphorylation of c-Jun at 24 h of differentiation (Supplementary Figures S1G–I). Thus, 24 h of differentiation was chosen as the sampling time to determine the combined effects of *Fzd7* knockdown, thermal stress, and growth selection on ROCK and c-Jun phosphorylation.

**FIGURE 2:**
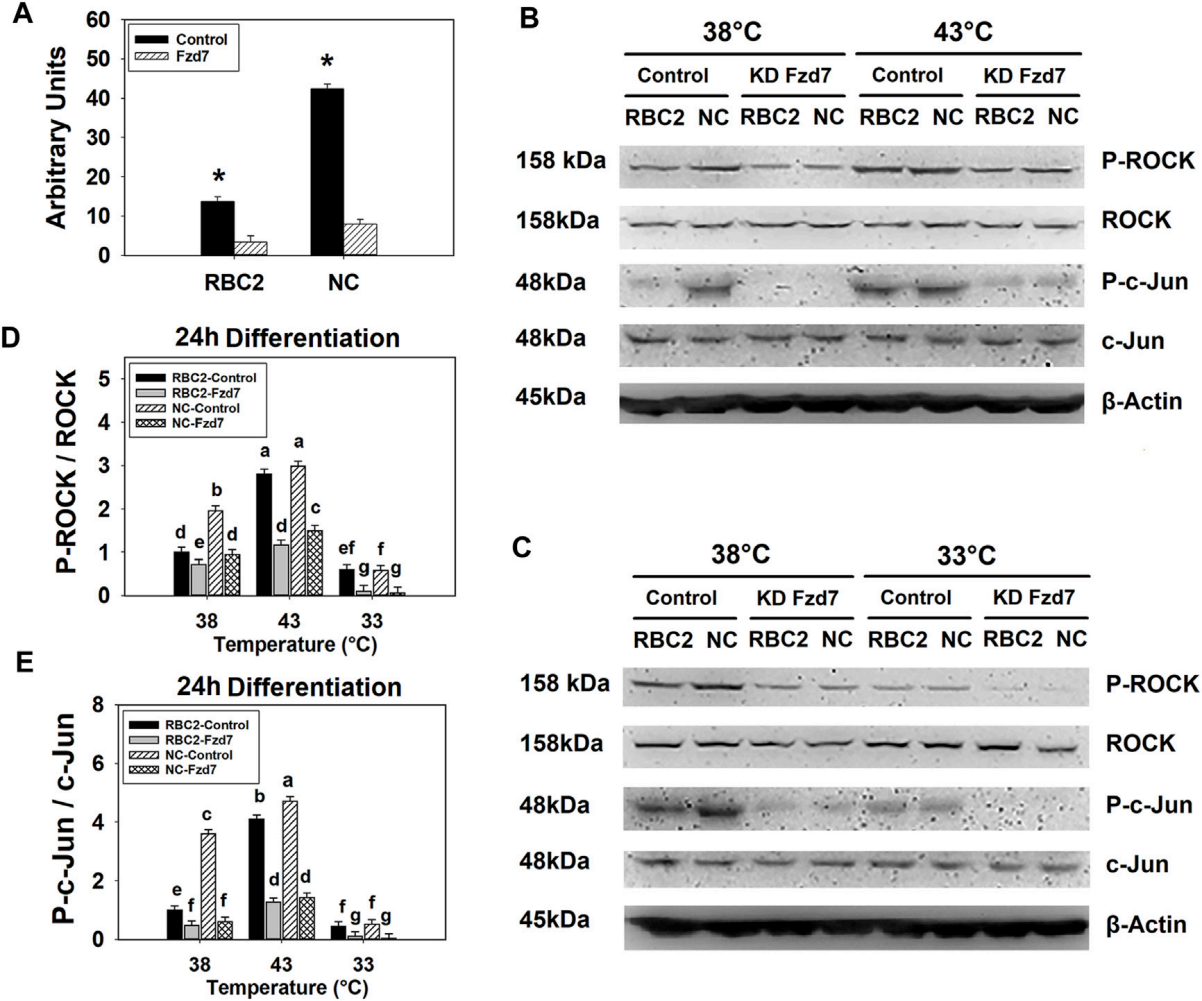
Knockdown of frizzled-7 (*Fzd7*) with small interfering RNA (siRNA) in satellite cells (SCs). **(A)** Expression of *Fzd7* mRNA was measured at 72 h of proliferation after *Fzd7* knockdown at the beginning of proliferation in Randombred Control Line 2 (RBC2) and modern commercial (NC) line SCs. Asterisk (*) above the bars represents a significant difference between the two adjacent groups (*p* ≤ 0.05). **(B)** Western blots of unphosphorylated and phosphorylated forms of rho-associated protein kinase (ROCK) and c-Jun, and an internal control β-actin in SCs from the RBC2 and NC lines cultured at 38°C and 43°C was determined at 24 h of differentiation. **(C)** Western blots of the unphosphorylated and phosphorylated forms of ROCK and c-Jun, and an internal control β-actin in SCs from the RBC2 and NC lines cultured at 38°C and 33°C was determined at 24 h of differentiation. **(D)** The ratio of phosphorylated to unphosphorylated ROCK. **(E)** The ratio of phosphorylated to unphosphorylated c-Jun. Treatment group was above of each lane, and molecular weight and protein name is on the left and right side of each figure in **(B and C)**. Control = SCs transfected with a negative control siRNA, and KD Fzd7 = SCs transfected with a siRNA targeting *Fzd7* in **(B and C)**. Each graph bar represents a mean ratio, and each error bar represents a standard error of the mean value. Mean values with different letters are significantly different (*p* ≤ 0.05).

Knockdown efficiency of *Fzd7* siRNA was determined in both lines at 38°C with RT-qPCR. At 72 h post transfection, mRNA expression of *Fzd7* decreased 4.08-fold (*p* < 0.001) and 5.31-fold (*p* < 0.001) in the RBC2 and NC *Fzd7* knockdown groups compared to the control groups, respectively (Figure 2A). At 24 h of differentiation, a significant interaction (*p* < 0.001) among the effects of *Fzd7* knockdown, temperature, and line was observed in phosphorylation of both ROCK and c-Jun (Figures 2B–E). Knockdown of *Fzd7* decreased phosphorylation of ROCK (*p* ≤ 0.013) and c-Jun (*p* < 0.001) in both lines at all the temperatures. However, the NC line showed greater reductions in phosphorylation of both ROCK and c-Jun compared to the RBC2 line independent of temperature.

### 3.3 Effect of Frizzled-7 Knockdown, Thermal Stress, and Growth Selection on Satellite Cell Proliferation

No significant interaction was observed among *Fzd7* knockdown, temperature, and line effects at any sampling time during SC proliferation (Table 2). A significant interaction was observed between temperature and cell line at 24 h (*p* = 0.003), 48 h (*p* < 0.001), and 72 h (*p* < 0.001). The factors of temperature and *Fzd7* knockdown significantly interacted at 72 h (*p* < 0.001). In both the RBC2 and NC control groups, heat stress (43°C) showed a positive effect on the proliferation of SCs (promote, *p* < 0.001) while cold stress (33°C) had a negative effect (suppress, *p* < 0.001) on proliferaton at 72 h. Knockdown of *Fzd7* decreased (*p* < 0.001) proliferation of both lines at 38°C (RBC2: 1.82-fold; NC: 1.70-fold) and 43°C (RBC2: 2.81-fold; NC: 1.58-fold) when measured at 72 h. At 33°C, no significant reduction in proliferation was observed in either line at any sampling time.

**TABLE 2:** Effect of temperature and knockdown of frizzled-7 (*Fzd7*) on proliferation of randombred control line 2 (RBC2) and modern commercial line (NC) satellite cells^1^.

Line	Temperature^2^	Knockdown^3^	Sampling time			
			0 h	24 h	48 h	72 h
RBC2	38	Control	0.14^a,x^ ± 0.07	0.20^c,x^ ± 0.08	0.44^cd,y^ ± 0.08	0.94^d,z^ ± 0.07
		Fzd7	0.13^a,x^ ± 0.09	0.15^cd,x^ ± 0.07	0.20^de,y^ ± 0.08	0.52^e,z^ ± 0.09
	43	Control	0.10^a,x^ ± 0.07	0.19^bc,x^ ± 0.08	0.51^c,y^ ± 0.07	1.55^c,z^ ± 0.06
		Fzd7	0.11^a,x^ ± 0.07	0.11^cd,x^ ± 0.07	0.24^cde,y^ ± 0.07	0.55^e,z^ ± 0.07
	33	Control	0.13^a,w^ ± 0.08	0.15^cd,x^ ± 0.09	0.17^de,y^ ± 0.07	0.23^f,z^ ± 0.08
		Fzd7	0.13^a,z^ ± 0.11	0.10^d,z^ ± 0.08	0.12^e,z^ ± 0.08	0.12^f,z^ ± 0.08
NC	38	Control	0.13^a,w^ ± 0.09	0.32^b,x^ ± 0.08	0.58^c,y^ ± 0.08	1.84^b,z^ ± 0.08
		Fzd7	0.13^a,x^ ± 0.08	0.21^c,x^ ± 0.08	0.41^cde,y^ ± 0.08	1.08^d,z^ ± 0.08
	43	Control	0.10^a,w^ ± 0.07	0.46^a,x^ ± 0.07	1.41^a,y^ ± 0.06	2.74^a,z^ ± 0.07
		Fzd7	0.10^a,x^ ± 0.07	0.27^b,x^ ± 0.07	0.97^b,y^ ± 0.06	1.73^bc,z^ ± 0.09
	33	Control	0.13^a,x^ ± 0.08	0.19^cd,y^ ± 0.07	0.23^de,y^ ± 0.07	0.34^ef,z^ ± 0.08
		Fzd7	0.11^a,x^ ± 0.07	0.15^cd,yx^ ± 0.09	0.19^de,y^ ± 0.09	0.32^ef,z^ ± 0.11
*p*-value^4^		L × T	0.999	0.003	<0.001	<0.001
		L × K	0.502	0.242	0.724	0.326
		T × K	0.747	0.176	0.125	<0.001
		L × T × K	0.664	0.528	0.589	0.099

1 Mean DNA concentration (μg/well) ± Standard error of mean (SEM).

2 Incubation temperature (°C).

3 Control, transfecting cells with a negative control small interfering RNA sequence; Fzd7, knockdown of Fzd7.

4 (L × T), interaction effect between line and temperature; (L × K), between line and knockdown; (T × K), between temperature and knockdown; (L × T × K), among line, temperature, and knockdown.

^a-f^Mean DNA concentration (μg/well ± SEM) within a column (sampling time) without a common letter are significantly different.

^w-z^Mean DNA concentration (μg/well ± SEM) within a row (line, temperature, and knockdown) without a common letter are significantly different.

p ≤ 0.05 was considered as significant different.

From 0 to 72 h of the proliferation assay, cell proliferation linearly increased as a function of sampling time in both the RBC2 and NC control groups at all the temperatures. The slope of the linear regression was always greater in the NC line compared to the RBC2 line (*p* ≤ 0.001). Knockdown of *Fzd7* decreased (*p* < 0.001) the regression slope in both lines at 38°C (RBC2: 2.20-fold; NC: 1.73-fold) and 43°C (RBC2: 3.18-fold; NC: 1.66-fold).

### 3.4 Effect of Frizzled-7 Knockdown, Thermal Stress, and Growth Selection on Satellite Cell Differentiation

#### 3.4.1 Knockdown of Frizzled-7 at Zero Hour of Proliferation

There was a significant interaction (*p* < 0.001) among the effects of *Fzd7* knockdown, temperature, and line at 0, 24, and 72 h of differentiation (Table 3). However, at 48 h, a significant interaction (*p* < 0.001) was only observed between line and temperature and between temperature and *Fzd7* knockdown. Within each temperature, differentiation of the NC control group was greater (*p* < 0.001) than that of the RBC2 control group at 0, 24 and 48 h of differentiation. In the control groups, differentiation was greater at 43°C (*p* ≤ 0.002), and less at 33°C (*p* ≤ 0.030) at all sampling times compared to 38°C. Differentiation was linearly increased in both the RBC2 and NC control groups from 0 to 48 h of differentiation at 38°C and 43°C. The NC control group had a greater slope as shown by linear regression compared to the RBC2 line only at 38°C (*p* = 0.003).

**TABLE 3:** Effect of temperature and knockdown of frizzled-7 (*Fzd7*) on differentiation of randombred control line 2 (RBC2) and modern commercial line (NC) satellite cells^1^.

Line	Temperature^2^	Knockdown^3^	Sampling time			
			0 h	24 h	48 h	72 h
RBC2	38	Control	5.40^c,w^ ± 1.34	12.46^e,x^ ± 1.13	49.45^c,y^ ± 1.34	67.62^b,z^ ± 1.34
		Fzd7	2.23^f,y^ ± 1.13	1.63^f,y^ ± 1.34	9.58^e,z^ ± 1.22	9.24^h,z^ ± 1.13
	43	Control	8.01^b,x^ ± 1.22	45.97^b,y^ ± 1.22	68.50^b,z^ ± 1.49	72.80^a,z^ ± 1.22
		Fzd7	4.36^cde,x^ ± 1.06	11.89^e,y^ ± 1.06	24.55^d,z^ ± 1.34	21.81^g,z^ ± 1.13
	33	Control	4.00^de,z^ ± 1.13	2.32^f,x^ ± 1.22	2.49^f,x^ ± 1.13	2.7^ij,y^ ± 1.22
		Fzd7	1.82^f,z^ ± 1.34	1.13^f,y^ ± 1.34	0.58^f,x^ ± 1.49	0.60^j,x^ ± 1.22
NC	38	Control	7.11^b,w^ ± 1.22	34.08^d,x^ ± 1.22	74.40^b,z^ ± 1.49	58.54^d,y^ ± 1.13
		Fzd7	4.86^cd,x^ ± 1.13	10.67^e,y^ ± 1.22	28.19^d,z^ ± 1.22	26.55^f,z^ ± 1.13
	43	Control	17.35^a,x^ ± 1.22	62.19^a,y^ ± 1.22	86.68^a,z^ ± 1.49	63.22^c,y^ ± 1.34
		Fzd7	7.99^b,w^ ± 1.22	37.19^c,x^ ± 1.49	49.49^c,z^ ± 1.22	44.48^e,y^ ± 1.22
	33	Control	4.40^cde,z^ ± 1.34	3.48^f,y^ ± 1.22	3.51^f,y^ ± 1.22	4.68^i,z^ ± 1.22
		Fzd7	3.33^e,z^ ± 1.13	1.91^f,yx^ ± 1.34	2.35^f,y^ ± 1.22	1.86^ij,x^ ± 1.49
*p*-value^4^		L × T	<0.001	<0.001	<0.001	0.008
		L × K	0.018	0.237	0.825	<0.001
		T × K	<0.001	<0.001	<0.001	<0.001
		L × T × K	<0.001	<0.001	0.121	<0.001

1 Mean creatine kinase activity (Unit/well) ± Standard error of mean (SEM).

2 Incubation temperature (°C).

3 Control, transfecting cells with a negative control small interfering RNA sequence; Fzd7, knockdown of Fzd7.

4 (L × T), interaction effect between line and temperature; (L × K), between line and knockdown; (T × K), between temperature and knockdown; (L × T × K), among line, temperature, and knockdown.

^a-f^Mean creatine kinase activity (Unit/well ± SEM) within a column (sampling time) without a common letter are significantly different.

^w-z^Mean creatine kinase activity (Unit/well ± SEM) within a row (line, temperature, and knockdown) without a common letter are significantly different.

p ≤ 0.05 was considered as significant different.

Knockdown of *Fzd7* decreased (*p* < 0.001) differentiation of SCs of both lines at 38°C and 43°C with a greater reduction observed in the RBC2 line compared to the NC line at all sampling times. At 33°C, knockdown of *Fzd7* caused no significant reduction at 24, 48 and 72 h. Differentiation changed linearly with the knockdown of *Fzd7* and the slope of the regression line decreased (*p* < 0.001) in both line at 38°C (RBC2: 6.05-fold; NC: 2.87-fold) and 43°C (RBC2: 3.14-fold; NC: 1.71-fold). The linear relationship between differentiation and sampling time was absent in either line at 33°C.

**FIGURE 3:**
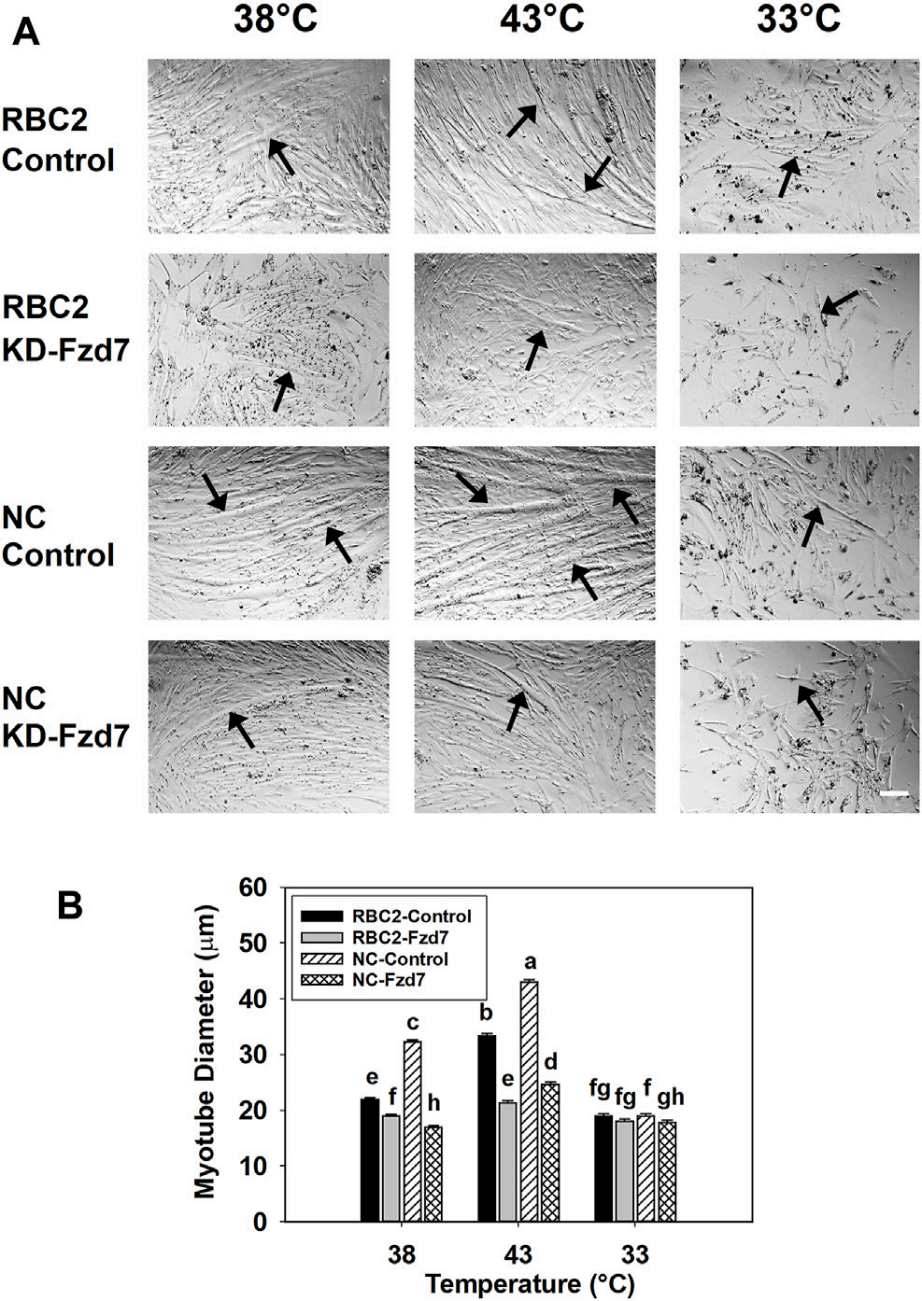
Effect of hot and cold thermal stress and knockdown of frizzled-7 (*Fzd7*) at the beginning of proliferation on myotube diameter. **(A)** Representative photomicrographs of Randombred Control Line 2 (RBC2) and modern commercial (NC) line SCs transfected with either a negative control siRNA (Control) or a siRNA targeting *Fzd7* (KD-Fzd7) at the beginning of proliferation and followed for 72 h of proliferation and 48 h of differentiation at 38°C, 43°C, or 33°C. **(B)** Diameter of myotubes in each treatment group. Each graph bar represents a mean diameter of the myotubes, and each error bar represents a standard error of the mean. Mean values with different letters are significantly different (*p* ≤ 0.05). Black arrows highlight representative myotubes. Scale bar (White) = 100 μm.

Knockdown of *Fzd7* also had a significant effect on myotube diameter. A significant interaction (*p* < 0.001) was observed among the effects *Fzd7* knockdown, temperature, and line at 48 h of differentiation (Figure 3). Myotube diameter increased with heat stress (43°C) and decreased with cold stress (33°C) in both line control groups. Knockdown of *Fzd7* decreased (*p* < 0.001) the myotube diameter in both lines at 38°C (RBC2: 1.16-fold; NC: 1.90-fold) and 43°C (RBC2: 1.56-fold; NC: 1.73-fold). At 33°C myotube diameter only decreased in the NC group (*p* = 0.040).

#### 3.4.2 Knockdown of Frizzled-7 at Zero Hour of Differentiation

The interaction among *Fzd7* knockdown, temperature, and line effect was significant (*p* < 0.001) at all sampling times (Table 4). At 38°C and 43°C, differentiation was greater in the NC line (*p* < 0.001) compared to the RBC2. Heat stress increased SC differentiation in both line at 0 and 72 h (*p* < 0.001), and cold stress showed a significant inhibitory effect (*p* < 0.001) on both lines at all sampling times. At 38°C, differentiation was lower (*p* < 0.001) in the RBC2 and NC *Fzd7* knockdown groups compared to the controls at 48 h (RBC2: 1.15-fold; NC: 1.14-fold) and 72 h (RBC2: 1.15-fold; NC: 1.08-fold). Knockdown of *Fzd7* also decreased (*p* < 0.001) SC differentiation at 43°C, with 1.21 and 1.46-fold reductions observed at 72 h in the RBC2 and NC lines, respectively. At 33°C, the knockdown effect was only significant in the RBC2 line at 72 h (*p* = 0.045). From 0 to 48 h, there was a linear increase in SC differentiation in the control and *Fzd7* knockdown groups of both lines at 38°C and 43°C. Knockdown of *Fzd7* at the beginning of differentiation did not significantly change the slope of the linear regression in either line at either 38°C or 43°C. A linear increase was absent in either line at 33°C.

**TABLE 4:** Effect of temperature and knockdown of frizzled-7 (*Fzd7*) at the beginning of differentiation on differentiation of randombred control line 2 (RBC2) and modern commercial line (NC) satellite cells^1^.

Line	Temperature^2^	Knockdown^3^	Sampling time			
			0 h	24 h	48 h	72 h
RBC2	38	Control	7.23^e,w^ ± 0.39	15.79^f,x^ ± 0.45	37.12^g,y^ ± 0.50	54.13^f,z^ ± 0.50
		Fzd7	6.39^ef,w^ ± 0.42	16.11^f,x^ ± 0.50	32.29^h,y^ ± 0.50	47.27^g,z^ ± 0.50
	43	Control	12.47^c,w^ ± 0.42	51.59^d,x^ ± 0.50	72.03^e,z^ ± 0.55	68.15^d,y^ ± 0.45
		Fzd7	10.79^d,w^ ± 0.42	43.66^e,x^ ± 0.45	60.97^f,z^ ± 0.55	56.37^e,y^ ± 0.55
	33	Control	5.54^fg,z^ ± 0.64	2.57^h,w^ ± 0.56	3.40^i,x^ ± 0.56	4.38^i,y^ ± 0.64
		Fzd7	4.65^g,z^ ± 0.56	2.16^h,x^ ± 0.56	3.45^i,y^ ± 0.79	2.52^j,x^ ± 0.56
NC	38	Control	11.83^c,w^ ± 0.42	68.78^a,x^ ± 0.50	111.20^a,z^ ± 0.55	83.74^b,y^ ± 0.55
		Fzd7	11.58^cd,w^ ± 0.50	54.4^c,x^ ± 0.55	97.81^b,z^ ± 0.50	77.33^c,y^ ± 0.50
	43	Control	24.75^a,w^ ± 0.45	57.87^b,x^ ± 0.50	85.66^c,y^ ± 0.45	99.01^a,z^ ± 0.55
		Fzd7	17.40^b,w^ ± 0.45	56.88^b,x^ ± 0.50	80.07^d,z^ ± 0.55	67.52^d,y^ ± 0.55
	33	Control	6.44^ef,y^ ± 0.79	4.70^g,x^ ± 0.64	4.27^i,x^ ± 0.64	8.11^h,z^ ± 0.64
		Fzd7	5.83^fg,z^ ± 0.64	4.43^g,y^ ± 0.79	3.75^i,y^ ± 0.79	6.53^h,z^ ± 0.64
*p*-value^4^		L × T	<0.001	<0.001	<0.001	<0.001
		L × K	<0.001	<0.001	0.214	<0.001
		T × K	<0.001	<0.001	<0.001	<0.001
		L × T × K	<0.001	<0.001	<0.001	<0.001

1 Mean creatine kinase activity (Unit/well) ± Standard error of mean (SEM).

2 Incubation temperature (°C).

3 Control, transfecting cells with a negative control small interfering RNA sequence; Fzd7, knockdown of Fzd7.

4 (L × T), interaction effect between line and temperature; (L × K), between line and knockdown; (T × K), between temperature and knockdown; (L × T × K), among line, temperature, and knockdown.

^a-f^Mean creatine kinase activity (Unit/well ± SEM) within a column (sampling time) without a common letter are significantly different.

^w-z^Mean creatine kinase activity (Unit/well ± SEM) within a row (line, temperature, and knockdown) without a common letter are significantly different.

p ≤ 0.05 was considered as significant different.

**FIGURE 4:**
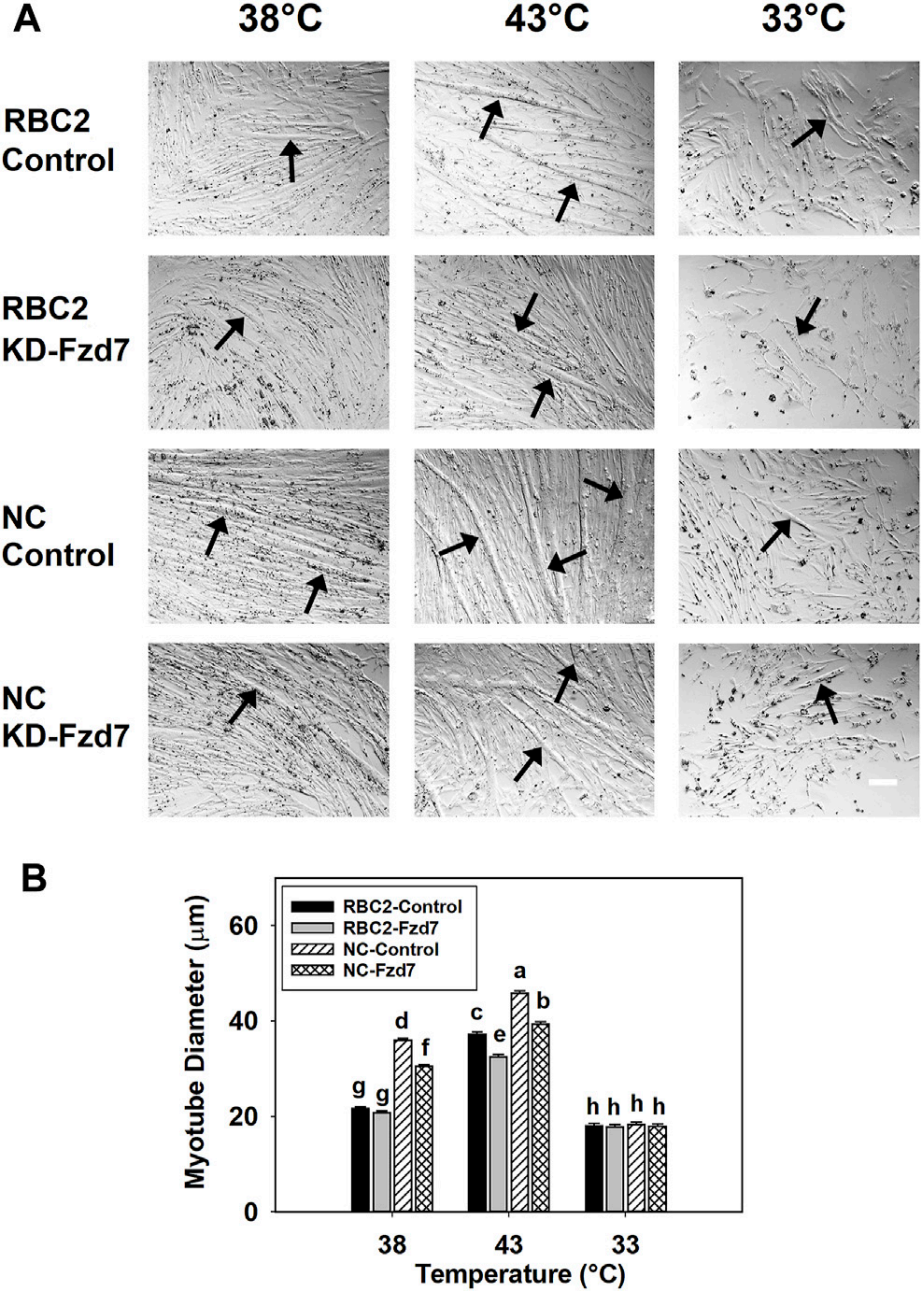
Effect of hot and cold thermal stress and knockdown of frizzled-7 (*Fzd7*) at the beginning of differentiation on myotube diameter. **(A)** Randombred Control Line 2 (RBC2) and modern commercial (NC) line SCs proliferated normally at 38°C, 43°C, or 33°C and transfected with either a negative control siRNA (Control) or a siRNA targeting *Fzd7* (KD-Fzd7) at 72 h of proliferation, and photomicrographs were captured at 48 h of differentiation. **(B)** Diameter of myotubes in each treatment group. Each graph bar represents a mean diameter of the myotubes, and each error bar represents a standard error of the mean. Mean values with different letters are significantly different (*p* ≤ 0.05). Black arrows highlight representative myotubes. Scale bar (White) = 100 μm.

Effects on myotube diameter were also seen when *Fzd7* was knocked down at the beginning of differentiation (Figure 4). The effects *Fzd7* knockdown, temperature, and line showed a significant (*p* < 0.001) interaction at 48 h of differentiation. Heat stress increased myotube diameter (*p* < 0.001) while cold stress decreased the diameter (*p* < 0.001) in both lines. However, at 38°C myotube diameter decreased only in the NC line, with the knockdown of *Fzd7* (1.18-fold, *p* < 0.001). Both lines had reductions (*p* < 0.001) in myotube diameter with the knockdown of *Fzd7* at 43°C (RBC2: 1.15-fold; NC: 1.16-fold). No significant knockdown effect was observed in either line at 33°C.

### 3.5 Effect of Frizzled-7 Knockdown, Thermal Stress, and Growth Selection on the Expression of Myogenic Regulatory Genes

#### 3.5.1 Myoblast Determination Factor 1 Expression

**FIGURE 5:**
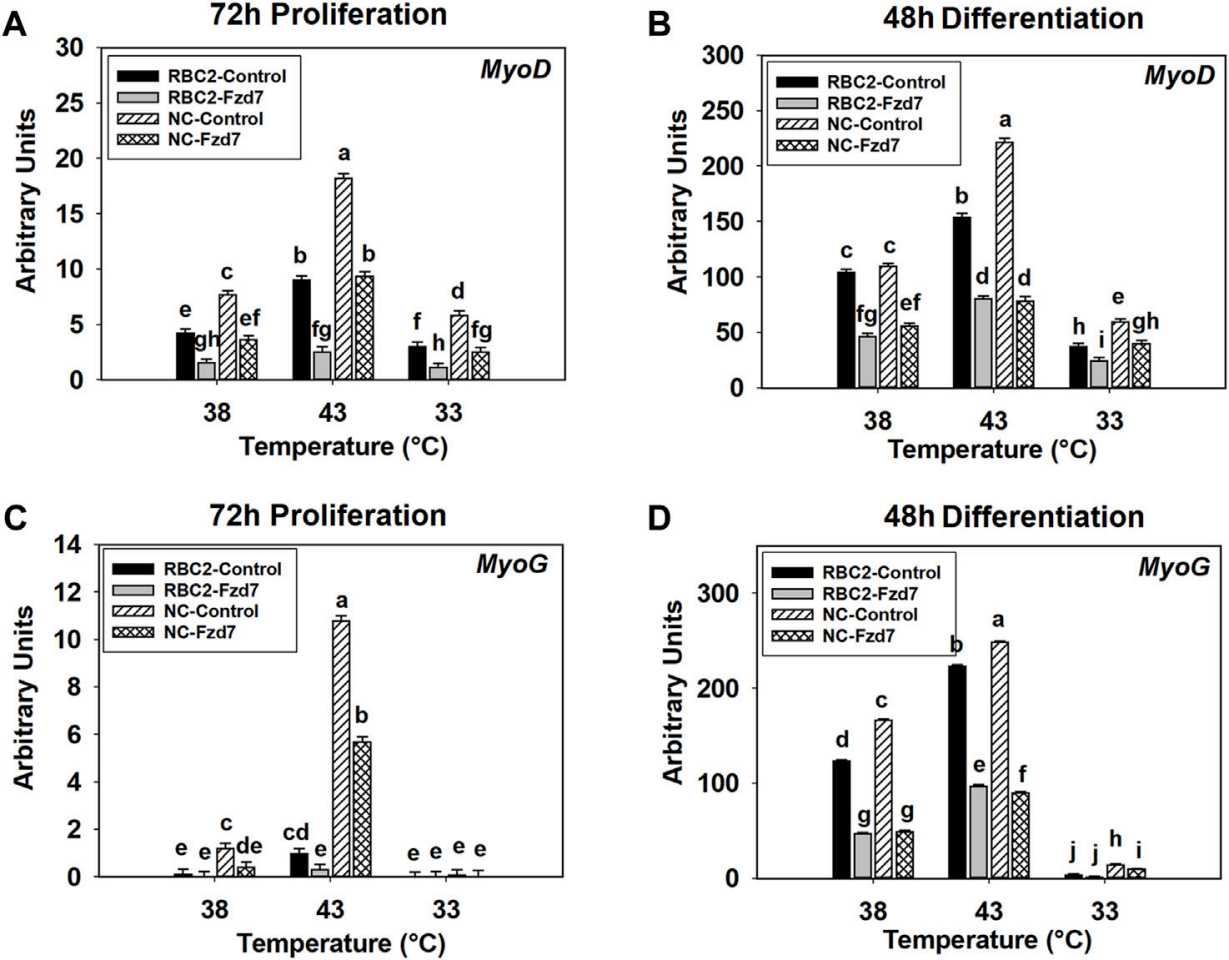
Effect of hot and cold thermal stress and frizzled-7 (*Fzd7*) knockdown on the expression of myogenic determination factor 1 (*MyoD*) and myogenin (*MyoG*) in satellite cells (SCs). After transfection with either a negative control siRNA or a siRNA targeting *Fzd7*, both Randombred Control Line 2 (RBC2) and modern commercial (NC) line SCs proliferated at 38°C, 43°C, or 33°C. Expression of *MyoD* was determined at 72 h of proliferation **(A)** and 48 h of differentiation **(B)**. Expression of *MyoG* was determined at 72 h of proliferation **(C)** and 48 h of differentiation **(D)**. Each graph bar represents a mean arbitrary unit, and each error bar represents a standard error of the mean. Mean values with different letters are significantly different (*p* ≤ 0.05).

Interaction effects were significant (*p* < 0.001) between temperature and line, between line and knockdown, and between temperature and knockdown at 72 h of proliferation (Figure 5A). Expression of *MyoD* increased at 43°C (*p* < 0.001) and decreased at 33°C (*p* < 0.001) in both lines compared to the control (38°C) (Figure 5A). Knockdown of *Fzd7* decreased (*p* < 0.001) *MyoD* expression in both lines, with the RBC2 line showing greater reductions (fold change) compared to the NC line at 33°C and 43°C (Figure 5A).

A significant three-way interaction was also observed among the effects of *Fzd7* knockdown, temperature, and line at 48 h of differentiation (Figure 5B). Heat stress (43°C) increased (*p* < 0.001) *MyoD* expression in both lines while cold stress (33°C) had an inhibitory effect (*p* < 0.001, Figure 5B). Knockdown of *Fzd7* reduced *MyoD* expression in both lines at all temperatures (Figure 5B).

#### 3.5.2 Myogenin Expression

A significant interaction was observed among *Fzd7* knockdown, temperature, and line effects at both 72 h of proliferation (*p* < 0.001, Figure 5C) and 48 h of differentiation (*p* < 0.001, Figure 5D). At 72 h of proliferation, heat stress (43°C) increased *MyoG* expression in both lines (*p* ≤ 0.004) while cold stress (33°C) showed no significant effect in either line (Figure 5C). Knockdown of *Fzd7* decreased expression of *MyoG* in the RBC2 and NC groups only at 43°C (Figure 5C).

At 48 h of differentiation, *MyoG* expression was increased at 43°C (*p* < 0.001) and decreased at 33°C (*p* < 0.001) in both lines (Figure 5D). Knockdown of *Fzd7* decreased *MyoG* expression (*p* < 0.001) in both lines at both 38°C and 43°C with the NC line having a greater reduction than the RBC2 line (Figure 5D). At 33°C, reduced *MyoG* expression was observed only in the NC *Fzd7* knockdown group compared to the control (*p* = 0.009, Figure 5D).

### 3.6 Effect of Frizzled-7 Knockdown, Thermal Stress, and Growth Selection on Satellite Cell Lipid Accumulation

#### 3.6.1 Lipid Accumulation During Proliferation

During proliferation, a significant interaction (*p* ≤ 0.046) for lipid accumulation was observed among *Fzd7* knockdown, temperature and line effects at all sampling times (Table 5). Lipid content increased (*p* < 0.001) with heat stress (43°C) and decreased (*p* < 0.001) with cold stress (33°C) in both line control groups at 48 and 72 h. At 48 and 72 h, the NC line had higher (*p* ≤ 0.007) lipid levels compared to the RBC2 line regardless of temperature. With knockdown of *Fzd7*, lipid content at 24, 48, and 72 h was significantly reduced (*p* ≤ 0.002) in both lines at all temperatures.

**TABLE 5:** Effect of temperature and knockdown of frizzled-7 (*Fzd7*) on lipid content during proliferation of randombred control line 2 (RBC2) and modern commercial line (NC) satellite cells^1^.

Line	Temperature^2^	Knockdown^3^	Sampling time			
			0 h	24 h	48 h	72 h
RBC2	38	Control	0.06^c,w^ ± 0.03	0.09^e,x^ ± 0.03	0.20^g,y^ ± 0.03	1.14^d,z^ ± 0.03
		Fzd7	0.05^d,x^ ± 0.03	0.07^f,y^ ± 0.03	0.04^j,w^ ± 0.03	0.28^ghi,z^ ± 0.03
	43	Control	0.06^c,x^ ± 0.03	0.09^de,x^ ± 0.03	0.80^c,y^ ± 0.03	3.42^b,z^ ± 0.03
		Fzd7	0.06^cd,x^ ± 0.03	0.04^h,x^ ± 0.03	0.15^h,y^ ± 0.03	0.61^f,z^ ± 0.03
	33	Control	0.06^cd,x^ ± 0.03	0.05^g,x^ ± 0.03	0.08^i,y^ ± 0.03	0.16^hi,z^ ± 0.04
		Fzd7	0.06^cd,y^ ± 0.03	0.03^h,x^ ± 0.03	0.01^j,w^ ± 0.03	0.12^i,z^ ± 0.03
NC	38	Control	0.11^b,w^ ± 0.03	0.20^a,x^ ± 0.03	0.96^b,y^ ± 0.03	2.32^c,z^ ± 0.03
		Fzd7	0.12^b,x^ ± 0.03	0.18^b,x^ ± 0.03	0.35^e,y^ ± 0.03	1.32^d,z^ ± 0.03
	43	Control	0.12^ab,x^ ± 0.02	0.14^c,x^ ± 0.03	1.42^a,y^ ± 0.03	4.56^a,z^ ± 0.03
		Fzd7	0.12^a,x^ ± 0.03	0.10^d,x^ ± 0.03	0.44^d,y^ ± 0.03	0.82^e,z^ ± 0.03
	33	Control	0.12^ab,x^ ± 0.03	0.10^d,x^ ± 0.03	0.28^f,y^ ± 0.03	0.49^fg,z^ ± 0.03
		Fzd7	0.12^ab,x^ ± 0.03	0.07^f,w^ ± 0.03	0.16^h,y^ ± 0.03	0.40^fgh,z^ ± 0.04
*p*-value^4^		L × T	<0.001	<0.001	<0.001	<0.001
		L × K	<0.001	0.502	<0.001	<0.001
		T × K	<0.001	<0.001	<0.001	<0.001
		L × T × K	<0.001	0.046	<0.001	<0.001

1 Mean of AdipoRed optical density (OD/well) ± Standard error of mean (SEM).

2 Incubation temperature during proliferation, °C.

3 Control, transfecting cells with a negative control small interfering RNA sequence; Fzd7, knockdown of Fzd7.

4 (L × T), interaction effect between line and temperature; (L × K), between line and knockdown; (T × K), between temperature and knockdown; (L × T × K), among line, temperature, and knockdown.

^a-h^Mean of AdipoRed OD (mean ± SEM) within a column (sampling time) without a common letter are significantly different.

^w-z^Mean of AdipoRed OD (mean ± SEM) within a row (line, temperature, and knockdown) without a common letter are significantly different.

p ≤ 0.05 was considered as significant different.

From 24 to 72 h of proliferation, lipid content in both lines linearly increased at all temperatures (Table 5). Within the control groups, the slope of the linear regression was larger in the NC line compared to the RBC2 line (*p* ≤ 0.023). Heat stress increased the slopes of both lines while cold stress decreased the slopes (*p* < 0.001). Knockdown of *Fzd7* decreased (*p* < 0.001) the slope of both lines at all temperatures [38°C (RBC2: 5.27-fold; NC: 1.92-fold), 43°C (RBC2: 6.09-fold; NC: 6.20-fold), and 33°C (RBC2: 1.28-fold; NC: 1.23-fold)].

#### 3.6.2 Lipid Accumulation During Differentiation

During differentiation, an interaction among the effects of *Fzd7* knockdown, temperature and line effects was significant (*p* < 0.001) at all sampling times (Table 6). Within the control groups, lipid content was greater at 43°C (*p* < 0.001) only in the NC line at 24 and 48 h. A significant decrease (*p* < 0.001) in lipid content was observed in both lines under cold stress (33°C) but only at 24 h of differentiation. Knocking down *Fzd7* significantly decreased (*p* ≤ 0.007) lipid content at 24 and 48 h in both lines at all temperatures, except at 33°C for the NC line (*p* = 0.247).

**TABLE 6:** Effect of temperature and knockdown of frizzled-7 (*Fzd7*) on lipid content during differentiation of randombred control line 2 (RBC2) and modern commercial line (NC) satellite cells^1^.

Line	Temperature^2^	Knockdown^3^	Sampling time		
			24 h	48 h	72 h
RBC2	38	Control	1.61^c,z^ ± 0.05	0.62^c,x^ ± 0.05	0.74^b,y^ ± 0.05
		Fzd7	0.09^e,x^ ± 0.05	0.25^e,z^ ± 0.05	0.18^f,y^ ± 0.05
	43	Control	1.65^c,z^ ± 0.05	0.44^d,y^ ± 0.05	0.25^e,x^ ± 0.05
		Fzd7	0.36^d,z^ ± 0.05	0.19^ef,z^ ± 0.05	0.21^ef,z^ ± 0.05
	33	Control	0.38^d,z^ ± 0.05	0.26^e,y^ ± 0.04	0.18^ef,x^ ± 0.05
		Fzd7	0.12^e,z^ ± 0.05	0.09^f,y^ ± 0.05	0.03^g,x^ ± 0.05
NC	38	Control	2.33^b,z^ ± 0.04	0.84^b,x^ ± 0.05	0.96^a,y^ ± 0.05
		Fzd7	1.50^c,z^ ± 0.05	0.38^d,x^ ± 0.06	0.59^c,y^ ± 0.05
	43	Control	2.65^a,z^ ± 0.04	1.88^a,y^ ± 0.05	0.90^a,x^ ± 0.04
		Fzd7	0.57^d,z^ ± 0.05	0.56^c,z^ ± 0.05	0.49^d,z^ ± 0.05
	33	Control	0.54^d,y^ ± 0.05	0.78^b,z^ ± 0.05	0.77^b,z^ ± 0.05
		Fzd7	0.41^d,z^ ± 0.05	0.37^d,z^ ± 0.05	0.15^f,y^ ± 0.05
*p*-value^4^		L × T	<0.001	<0.001	<0.001
		L × K	0.921	<0.001	<0.001
		T × K	<0.001	<0.001	<0.001
		L × T × K	<0.001	<0.001	<0.001

1 Mean of AdipoRed optical density (OD/well) ± Standard error of mean (SEM).

2 Incubation temperature during differentiation, °C.

3 Control, transfecting cells with a negative control small interfering RNA sequence; *Fzd7*, knockdown of *Fzd7*.

4 (L × T), interaction effect between line and temperature; (L × K), between line and knockdown; (T × K), between temperature and knockdown; (L × T × K), among line, temperature, and knockdown.

^a-g^Mean of AdipoRed OD (mean ± SEM) within a column (sampling time) without a common letter are significantly different.

^x-z^Mean of AdipoRed OD (mean ± SEM) within a row (line, temperature, and knockdown) without a common letter are significantly different.

*p* ≤ 0.05 was considered as significant different.

From 24 to 72 h of differentiation, lipid content showed a linear decrease at 43°C in the control groups for both lines (Table 6). The slope of the linear regression (*p* < 0.001) was negative [9.13-fold and 22.75-fold in the RBC2 and NC *Fzd7* (*p* < 0.001) knockdown groups, respectively]. At 38°C and 33°C, lipid content was lower (*p* < 0.001) in both line control groups s at 48 and 72 h, compared to the 24 h.

### 3.7 Effect of Frizzled-7 Knockdown, Thermal Stress, and Growth Selection on the Expression of Adipogenic Regulatory Genes

#### 3.7.1 Peroxisome Proliferator Activated Receptor-Gamma Expression

**FIGURE 6:**
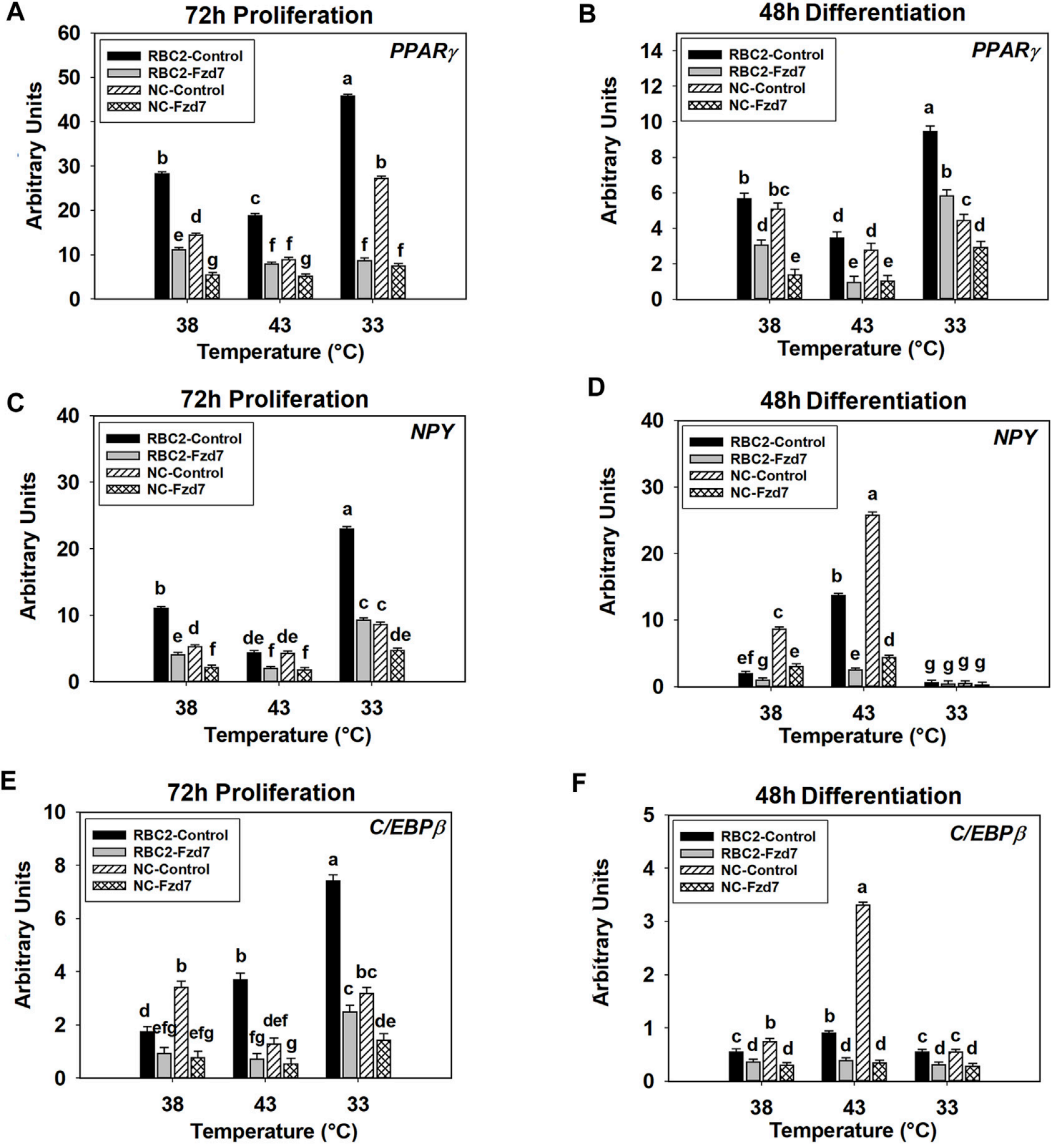
Effect of hot and cold thermal stress and frizzled-7 (*Fzd7*) knockdown on the expression of proliferator-activated receptor gamma (*PPARγ*), neuropeptide-Y (*NPY*), and CCAAT/Enhancer-Binding Protein-Beta (*C/EBPβ*) in satellite cells (SCs). After transfection with either a negative control siRNA or a siRNA targeting *Fzd7*, both Randombred Control Line 2 (RBC2) and modern commercial (NC) line SCs proliferated at 38°C, 43°C, or 33°C. Expression of *PPARγ* was determined at 72 h of proliferation **(A)** and 48 h of differentiation **(B)**. Expression of *NPY* was determined at 72 h of proliferation **(C)** and 48 h of differentiation **(D)**. Expression of *C/EBPβ* was determined at 72 h of proliferation **(E)** and 48 h of differentiation **(F)**. Each graph bar represents a mean arbitrary unit, and each error bar represents a standard error of the mean. Mean values with different letters are significantly different (*p* ≤ 0.05).

A significant interaction was observed among the effects *Fzd7* knockdown, temperature, and line at both 72 h of proliferation (*p* < 0.001, Figure 6A) and 48 h of differentiation (*p* < 0.001, Figure 6B). With heat stress (43°C), *PPARγ* expression decreased (*p* < 0.001) in both lines with a greater reduction observed in the NC line compared to the RBC2 line at 72 h of proliferation (Figure 6A). In contrast, expression of *PPARγ* was greatly increased with the cold stress (33°C), with the NC line showing a greater increase (Figure 6A). At 48 h of differentiation, *PPARγ* expression increased (*p* < 0.001) in both lines at 43°C but decreased at 33°C in the RBC2 line (*p* < 0.001) (Figure 6B). Knockdown of *Fzd7* downregulated (*p* ≤ 0.002) expression of *PPARγ* in both lines at both sampling times, with greater reductions (fold change) in the RBC2 line compared to the NC line at both 43°C and 33°C (Figures 6A,B).

#### 3.7.2 Neuropeptide-Y Expression

The effects of *Fzd7* knockdown, temperature, and line significantly interacted with each other at both sampling times (*p* < 0.001, Figures 6C,D). At 72 h of proliferation, heat stress (43°C) inhibited (*p* < 0.001) *NPY* expression in the RBC2 control group while cold stress (33°C) had a positive effect (*p* < 0.001, Figure 6C). In the NC control group, *NPY* expression showed a significant positive response to the cold stress (*p* < 0.001, Figure 6C). With the knockdown of *Fzd7*, *NPY* expression was downregulated (*p* < 0.001) in both lines at all temperatures during proliferation with the RBC2 line having greater reductions compared to the NC line at 38°C and 33°C (Figure 6C). At 48 h of differentiation, expression of *NPY* was upregulated at 43°C (*p* < 0.001) and downregulated at 33°C (*p* ≤ 0.013) in both the RBC2 and NC compared to 38°C (*p* < 0.001, Figure 6D). Knocking down *Fzd7* inhibited *NPY* expression in both lines at 38°C and 43°C (*p* < 0.001), with the NC SCs showing greater reductions than the RBC2 SCs (Figure 6D).

#### 3.7.3 CCAAT/Enhancer-Binding Protein-Beta Expression

There was a significant interaction among the *Fzd7* knockdown, temperature, and line effects during both proliferation and differentiation (Figures 6E,F). At 72 h of proliferation, *C/EBPβ* expression increased (*p* < 0.001) in the RBC2 control group at both 43°C and 33°C (Figure 6E). The NC SCs showed reduced *C/EBPβ* expression only at 43°C (*p* < 0.001) (Figure 6E). Knockdown of *Fzd7* suppressed expression of *C/EBPβ* at all temperatures (*p* < 0.001, Figure 5E). The RBC2 line had a greater reduction in C/EBPβ expression than the NC line at 43°C and 33°C (Figure 5E). At 48 h of differentiation, heat stress upregulated the expression of *C/EBPβ* in both the RBC2 and NC controls (*p* < 0.001), while cold stress downregulated the *C/EBPβ* only in the NC control group (*p* = 0.027, Figure 6F). Expression of *C/EBPβ* was significant lower (*p* ≤ 0.019) in both the RBC2 and NC *Fzd7* knockdown groups, compared to the controls at all temperatures with the NC line having greater reductions than the RBC2 line (Figure 6F).

## 4 Discussion

**FIGURE 7:**
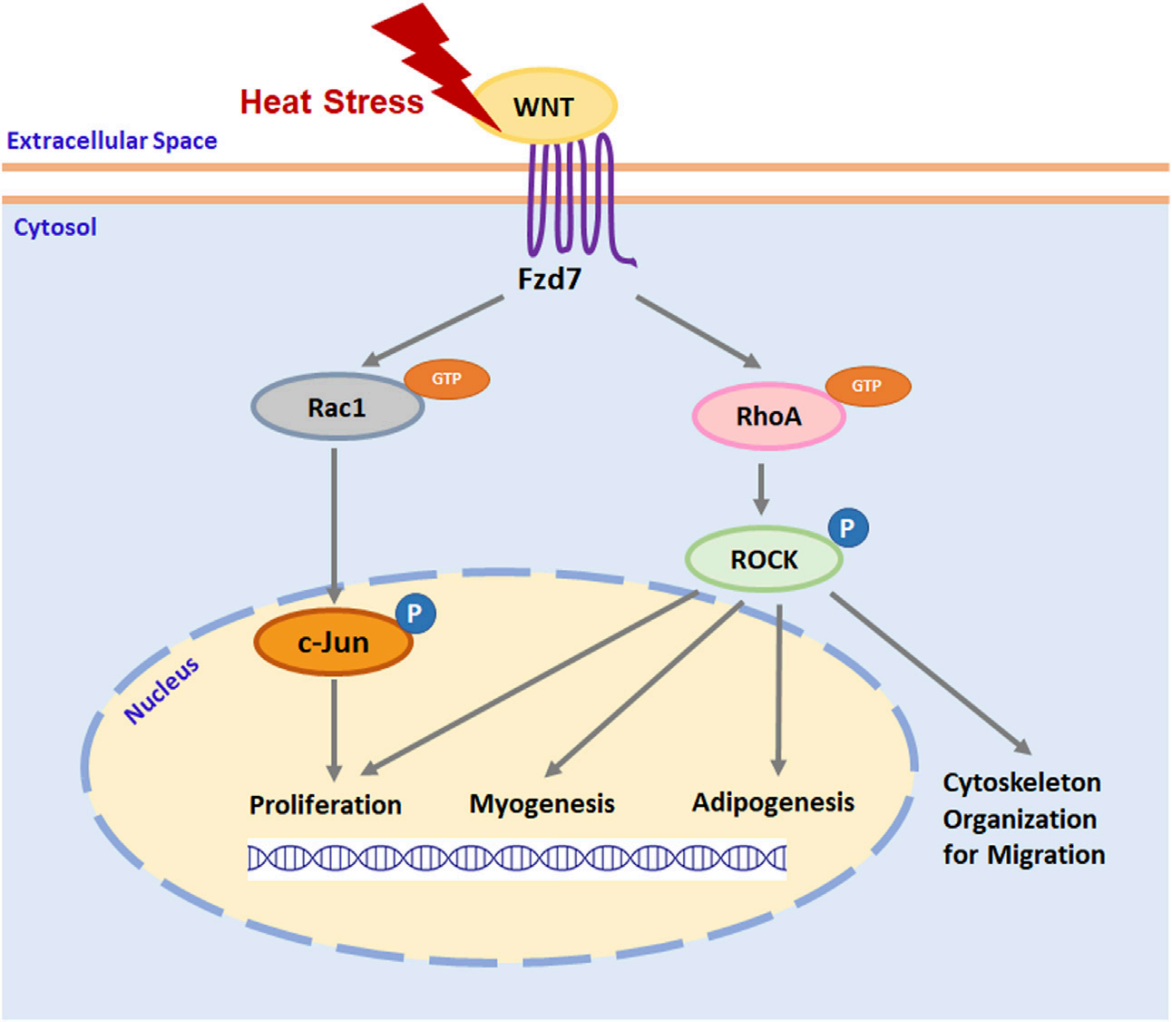
A schematic illustration of thermal stress regulation of satellite cell function and fate through Wnt planar cell polarity pathway. Heat stress stimulates the expression of wingless-type mouse mammary tumor virus integration site family (Wnt) protein ligands. Interaction between Wnt ligand and cell surface receptor Frizzled-7 (Fzd7) stimulates the activation of ras homolog gene family member A (RhoA) and ras-related C3 botulinum toxin substrate 1 (Rac1) by binding guanosine-5′-triphosphate (GTP) to RhoA and Rac1. Activated RhoA and Rac1 will activate its downstream effector rho-associated protein kinase (ROCK) and c-Jun, respectively, by phosphorylation (P). Activated ROCK not only organizes cytoskeleton protein for cell migration but also regulates the expression of specific genes that are associated with satellite cell proliferation, myogenesis, and adipogenesis. Activated c-Jun regulates satellite cell proliferation by stimulating the expression of genes promoting cell cycle progression through the G1 phase.

Thermal stress immediately after hatch has been shown to affect the growth and structure of the poultry p. major muscle (Piestun et al., 2017; Patael et al., 2019; Halevy, 2020), in part, through altering proliferation (Halevy et al., 2001; Xu et al., 2021b), myogenic differentiation (Halevy et al., 2001; Xu et al., 2021b), and adipogenic potential (Xu et al., 2021a) of SCs. Selection for growth further affects the function and fate of poultry p. major muscle SCs (Clark et al., 2016; Clark et al., 2017; Xu et al., 2021a; Xu et al., 2021b). As shown by transcriptome analysis, the Wnt pathway is greatly altered by thermal stress in turkey p. major muscle SCs (Reed et al., 2017a). Among the most affected *Wnt* genes, *Wnt7a*, which regulates the Wnt/PCP pathway through the Fzd7 receptor (Le Grand et al., 2009; Von Maltzahn et al., 2012), was significantly upregulated during heat stress, particularly in SCs from a growth-selected turkey line (Reed et al., 2017a). In mouse myoblasts, expression of *Wnt7a* and *Fzd7* increases with elevated temperatures (Risha et al., 2021). The Wnt7a-Fzd7-initiated Wnt/PCP pathway has been shown to stimulate the proliferation of SCs, promote myofiber hypertrophy, and increase skeletal muscle mass in mammals (Le Grand et al., 2009). Furthermore, the Wnt/PCP pathway is also involved in regulating the migration (Wang et al., 2018), myogenic differentiation (Iwasaki et al., 2008), and adipogenesis (Sordella et al., 2003; Bryan et al., 2005; Hosoyama et al., 2009) in mouse myoblasts through downstream RhoA/ROCK signaling and stimulates myoblast proliferation *via* Rac1/c-Jun signaling (Umansky et al., 2015; Hindi and Kumar, 2016). A schematic illustration of how thermal stress may affect satellite cell function and fate through the Fzd7-mediated Wnt/PCP pathway is presented in Figure 7. Taken together, these studies suggest both thermal stress and growth selection may alter poultry breast muscle growth, structure, and protein to fat ratio through a SC-mediated Wnt/PCP-dependent mechanism.

Satellite cells comprise a heterogeneous population (Schultz, 1974; Kuang et al., 2007; Tierney and Sacco, 2016) of multipotential stem cells (Asakura et al., 2001; Shefer et al., 2004). Selection for increased breast muscle yield in the turkey (Havenstein et al., 2003) has facilitated the conversion of the SCs in the p. major muscle to a population with higher rates of proliferation and myogenic differentiation (Velleman et al., 2000; Clark et al., 2016; Xu et al., 2021b) and elevated adipogenic potential (Velleman, 2014; Xu et al., 2021a). Results from the current study support these findings as the commercial NC line SCs showed significant increases in proliferation, myogenic differentiation, myotube diameter, and lipid accumulation compared to the non-selected RBC2 line. Phosphorylation (activation) of both ROCK and c-Jun was greater in the NC line compared to the RBC2 line. The activity of ROCK is associated with myoblast migration (Wang et al., 2018), myogenic differentiation (Iwasaki et al., 2008), and adipogenesis (Hosoyama et al., 2009) while c-Jun regulates the proliferation of SCs (Hindi and Kumar, 2016). With increased Wnt/PCP pathway signal transduction as supported by increased ROCK and c-Jun activity, the commercial NC line SCs can create a larger SC pool having a higher myogenic potential for myofiber hypertrophy compared to the RBC2 line. As reported by Xu et al. (2021a), the NC line SCs also synthesize more lipid than the RBC2 line during late proliferation and early differentiation, when SCs are not fully committed to a myogenic pathway. These studies suggest growth selection may have increased the myofiber hypertrophy and fat deposition in the turkey p. major muscle through a SC-mediated Wnt/PCP-dependent mechanism.

Post-hatch thermal stress appears to affect myofiber hypertrophy and fat deposition in the p. major muscle by regulating the Wnt/PCP pathway. Previous studies have reported expression of *Wnt7a* was greatly upregulated during heat stress in both mouse myoblasts (Risha et al., 2021) and turkey SCs (Reed et al., 2017a). The activity of SCs including proliferation and myogenic differentiation (Clark et al., 2016; Xu et al., 2021b) as well as the lipid synthesis (Clark et al., 2017; Xu et al., 2021a) was also increased during heat stress. In the current study, heat stress not only stimulated the activity of SCs but also promoted phosphorylation of both ROCK and c-Jun. Inhibition of the Wnt/PCP pathway through knockdown of *Fzd7* suppressed proliferation, differentiation, and adipogenesis of SCs during heat stress. These findings imply that heat stress-induces the Wnt/PCP pathway and may change the structure of turkey p. major muscle by altering the function and fate of SCs. Growth-selected faster-growing turkeys can display impaired p. major muscle morphology including decreased connective tissue spacing, reduced capillary density, and myofiber degeneration at normal growth temperature (Velleman et al., 2003). As homeotherms, chickens and turkeys have a reduced capacity to maintain body temperature (Yahav, 2000; Yahav, 2015). Fast-growing chickens exposed to heat stress also have lower capillary density (Hadad et al., 2014; Joiner et al., 2014), increased myofiber degeneration (Joiner et al., 2014), and more fat depots (Zhang et al., 2012) in the p. major muscle compared to heat-stressed slowing-growing chickens. Thus, environmental heat stress can further affect the development of muscle structure.

Cold stress, in contrast, has inhibitory effects on the activities of turkey SCs including proliferation and myogenic differentiation (Clark et al., 2016; Xu et al., 2021b) and lipid synthesis (Xu et al., 2021a), and the SCs of growth-selected turkeys exhibit a lower tolerance to cold stress compared to non-select turkeys. Reed et al. (2017a) reported that a greater number of genes significantly changed during cold stress in SCs from a growth-selected turkey line compared to that of a non-selected line. In the present study, both the Wnt/PCP pathway, as shown by the phosphorylation of ROCK and c-Jun, and SC activity as reflected by proliferation, myogenic differentiation, and lipid accumulation were suppressed during cold stress. This is similar to the response of mouse myoblasts where cold stress downregulated the expression of *Wnt7a* and *Fzd7* (Risha et al., 2021). These findings suggest that cold stress has an inhibitory effect on turkey breast muscle growth and structure, in part, by suppressing the Fzd7-mediated Wnt/PCP pathway, and that fast-growing turkeys are more sensitive to cold stress.

With regard to SC proliferation, the mitotic activity of poultry SCs peaks during the first week after hatch (Mozdziak et al., 1994; Halevy et al., 2000). During this time, SCs are highly responsive to temperature (Halevy et al., 2001; Halevy, 2020) as evidenced by increased proliferation and expression of *MyoD* with heat stress and suppression under cold stress (Clark et al., 2016; Xu et al., 2021b). Inhibition of the Wnt/PCP pathway through knockdown of *Fzd7* significantly decreased both the rate of proliferation and *MyoD* expression in both lines at both the control (38°C) and elevated (43°C) temperatures. The effect of the knockdown was not significant under cold stress since the proliferation of SCs had already been suppressed. These results imply that the Wnt/PCP pathway through the Fzd7 receptor acts in the proliferation of turkey SCs only at higher temperatures. The SCs of the growth-selected NC line were less dependent on the Fzd7-mediated Wnt/PCP pathway in maintaining proliferation compared to the RBC2 line. It is possible that other signaling pathways which support the proliferation of SCs have also been affected by growth selection. As reported by Xu et al. (2022a), the mechanistic target of rapamycin (mTOR) signaling pathway, which also stimulates avian SC proliferation (Vignale et al., 2015; Xu et al., 2022a), has increased activity in the NC line compared to the RBC2 line independent of temperature. With more proliferation-promotive pathways being activated, SCs from faster-growing turkeys are more likely to create a larger SC pool, and thus promoting breast muscle mass accretion through hypertrophic growth.

With the progression of myogenic differentiation, SCs in cell cultures gradually fuse to form multinucleated myotubes (McFarland et al., 1988). The activity of both ROCK and c-Jun in the turkey SCs was higher during the early stages of differentiation and gradually decreased. This can be expected as the function of ROCK and c-Jun in myogenic cells is dependent on cellular developmental stage. Both ROCK (Castellani et al., 2006) and c-Jun (Umansky et al., 2015; Hindi and Kumar, 2016) function in maintaining the proliferation of myogenic cells. During the early stages of myogenic differentiation, migration is required for the alignment of SCs before fusion to form multinucleated myotubes (Chazaud et al., 1998). The activation of ROCK regulates the migration of myogenic cells (Fortier et al., 2008; Wang et al., 2018), and therefore, is required during early differentiation. In the current study, knockdown of *Fzd7* decreased the activity of both ROCK and c-Jun. If *Fzd7* knockdown occurred at the beginning of proliferation, SC differentiation as well as myotube diameter was reduced to a greater extent compared to *Fzd7* being knocked down at the beginning of differentiation. This suggests that the Fzd7-mediated Wnt/PCP pathway affects myogenic differentiation of SCs mainly through regulating SC proliferation and migration prior to subsequent fusion to existing myofibers.

Satellite cell adipogenic potential depends on the developmental stage of the p. major muscle SCs (Xu et al., 2021a). Due to asymmetric division of SCs (Shinin et al., 2006), some daughter cells self-renew to maintain the SC pool (Kuang et al., 2007), some commit to a myogenic pathway (Kuang et al., 2007), while others spontaneously convert to an adipogenic population (Rossi et al., 2010). The lipid content in turkey SCs peaks during later proliferation and early differentiation (Xu et al., 2021a). Thus, the adipogenic potential of SCs is higher when SC fate is not fully committed to form a differentiated myotube. Furthermore, SC adipogenesis is also growth-dependent with the faster-proliferating population synthesizing more lipids than the slower-proliferating population (Rossi et al., 2010; Xu et al., 2021a). After the SCs begin differentiating, adipogenic potential will gradually decrease as reflected in the linear reduction in lipid content. Knockdown of *Fzd7* significantly decreased both lipid accumulation and the expression of *PPARγ*, *C/EBPβ*, and *NPY* during later proliferation and early differentiation. The commercial NC line SCs were less dependent on the Fzd7-mediated Wnt-PCP pathway in maintaining lipid accumulation compared to the RBC2 line. Together, these findings suggest thermal stress-induced changes in SC adipogenesis is regulated, in part, by the Fzd7-mediated Wnt/PCP pathway in a developmental time- and growth-dependent manner.

Additional pathways associated with adipogenesis may also be affected by growth selection. For example, activity of the mTOR pathway is higher in the NC line SCs compared to the RBC2 line independent of temperature (Xu et al., 2022a). The increased mTOR activity stimulates the adipogenesis in both mammalian (Yue et al., 2010) and avian (Xu et al., 2022b) SCs. Changes in SC adipogenesis *in vivo* is associated with altered intramuscular fat deposition (Piestun et al., 2017; Patael et al., 2019). Heat stress during the period of SC peak mitotic activity, the first week after hatch, may greatly increase the fat deposition in the p. major muscle of faster-growing turkeys. Increased intramuscular fat deposition may be associated with fat-related myopathies like white striping in the turkey p. major muscle (Soglia et al., 2018; Zampiga et al., 2019), and result in reduced breast meat quality.

In conclusion, the results from the current study indicate that thermal stress affects proliferation, differentiation, lipid accumulation, and expression of myogenic and adipogenic regulatory genes in turkey p. major muscle SCs through the Fzd7-mediated Wnt/PCP pathway in a growth-dependent manner. Specifically, heat stress promotes proliferation, differentiation, and lipid synthesis by stimulating the phosphorylation of both ROCK and c-Jun in both the NC and RBC2 line SCs, while cold stress showed an inhibitory effect. During cold stress, knockdown of the expression of *Fzd7* suppressed the proliferation, differentiation, and lipid accumulation in both lines of SCs. The reduction in these processes were greater in the commercial NC line compared to the non-selected RBC2 line. At normal temperature or during heat stress, the proliferation, differentiation, and adipogenesis of the NC line SCs, were overall less responsive to the knockdown of *Fzd7* compared to the non-selected RBC2 line. Furthermore, knockdown of *Fzd7* at the beginning of proliferation decreased the differentiation and myotube diameter to a greater extent in both lines compared to the knockdown of *Fzd7* at the beginning of differentiation. These results indicate that Fzd7-mediated SC myogenesis was initiated during proliferation. Hence, thermal stress during the period of SC peak mitotic activity may affect the structure and composition of turkey p. major muscle, in part, through the Fzd7-mediated Wnt/PCP pathway. During heat stress, increased SC proliferation and differentiation is associated with myofiber hypertrophy whereas intracellular lipid production will result in more intramuscular fat depots. Muscle fibers resulting from excessive hypertrophic growth will occupy the available connective tissue spacing and come in contact with each other (Velleman et al., 2003). This will result in degeneration of the muscle fibers. Furthermore, increased intramuscular fat depots may change the protein to fat ratio and affect turkey breast meat quality. Future studies will need to assess mechanisms of other signal transduction pathways in mediating SC function and fate. Target pathways include NPY- and myocyte enhancer factor 2C (MEF2C)-related signal transduction as expression of *NPY* (Reed et al., 2017b; Clark et al., 2018) and *MEF2C* (Reed et al., 2017b) are altered by thermal stress and growth selection in turkey SCs.

## Data Availability

The original contributions presented in the study are included in the article/Supplementary Material, further inquiries can be directed to the corresponding author.
