# Supplementary figures and images for: Temperature and Growth Selection Effects on Proliferation, Differentiation, and Adipogenic Potential of Turkey Myogenic Satellite Cells Through Frizzled-7-Mediated Wnt Planar Cell Polarity Pathway
Source: Front Physiol. 2022 May 23;13:892887. doi: 10.3389/fphys.2022.892887 (PMC9167958; doi:10.3389/fphys.2022.892887)

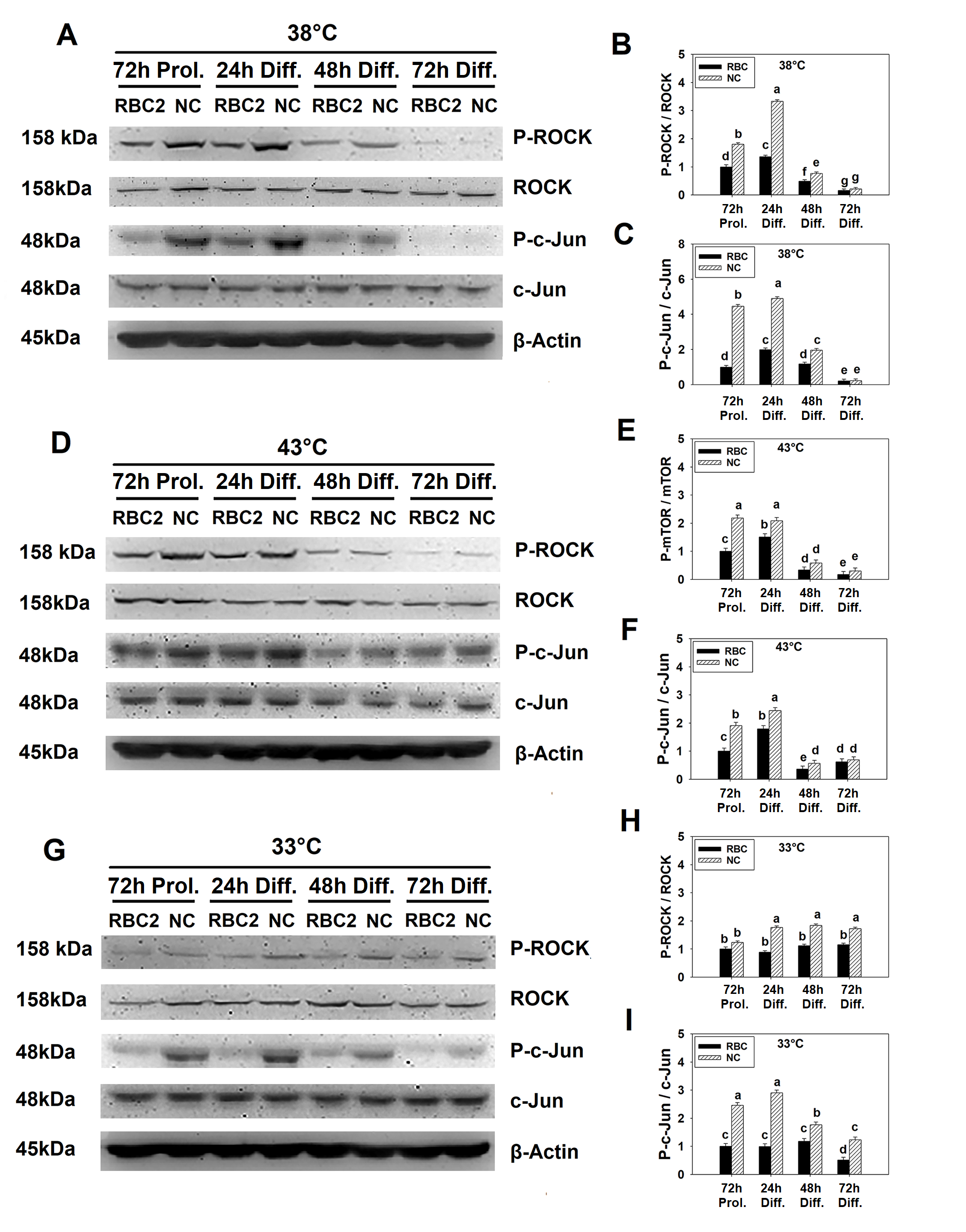

Supplement: Supplementary file 1 [file Image1.TIF]
